# Designing Symmetric Gradient Honeycomb Structures with Carbon-Coated Iron-Based Composites for High-Efficiency Microwave Absorption

**DOI:** 10.1007/s40820-024-01435-z

**Published:** 2024-07-02

**Authors:** Yu Zhang, Shu-Hao Yang, Yue Xin, Bo Cai, Peng-Fei Hu, Hai-Yang Dai, Chen-Ming Liang, Yun-Tong Meng, Ji-Hao Su, Xiao-Juan Zhang, Min Lu, Guang-Sheng Wang

**Affiliations:** 1 School of Chemistry, Beihang University, Beijing, 100191 People’s Republic of China; 2 School of Electronics and Information, Zhengzhou University of Light Industry, Zhengzhou, 450002 People’s Republic of China; 3 School of Chemical Engineering, Northeast Electric Power University, Jilin, 132000 People’s Republic of China; 4 College of Chemistry and Materials Engineering, Beijing Technology and Business University, Beijing, 100048 People’s Republic of China

**Keywords:** MIL-88C (Fe), Fe/Fe_3_O_4_/Fe_3_C@C, Controllable preparation, Symmetric gradient honeycomb structure, Microwave absorbing

## Abstract

The impedance matching of absorbers is a vital factor affecting their microwave absorption (MA) properties. In this work, we controllably synthesized Material of Institute Lavoisier 88C (MIL-88C) with varying aspect ratios (AR) as a precursor by regulating oil bath conditions, followed by one-step thermal decomposition to obtain carbon-coated iron-based composites. Modifying the precursor MIL-88C (Fe) preparation conditions, such as the molar ratio between metal ions and organic ligands (M/O), oil bath temperature, and oil bath time, influenced the phases, graphitization degree, and AR of the derivatives, enabling low filler loading, achieving well-matched impedance, and ensuring outstanding MA properties. The MOF-derivatives 2 (MD_2_)/polyvinylidene Difluoride (PVDF), MD_3_/PVDF, and MD_4_/PVDF absorbers all exhibited excellent MA properties with optimal filler loadings below 20 wt% and as low as 5 wt%. The MD_2_/PVDF (5 wt%) achieved a maximum effective absorption bandwidth (EAB) of 5.52 GHz (1.90 mm). The MD_3_/PVDF (10 wt%) possessed a minimum reflection loss (*RL*_min_) value of − 67.4 at 12.56 GHz (2.13 mm). A symmetric gradient honeycomb structure (SGHS) was constructed utilizing the high-frequency structure simulator (HFSS) to further extend the EAB, achieving an EAB of 14.6 GHz and a *RL*_min_ of − 59.0 dB. This research offers a viable inspiration to creating structures or materials with high-efficiency MA properties.

**Supplementary Information:**

The online version contains supplementary material available at 10.1007/s40820-024-01435-z.

## Highlights

- MIL-88C (Fe) with varying aspect ratios as a precursor was synthesized by regulating oil bath conditions, followed by one-step thermal decomposition to obtain carbon-coated iron-based composites.
- High-efficiency microwave absorption properties were achieved with *RL*_min_ value of -67.4 dB (2.13 mm) and wide effective absorption bandwidth (EAB) of 5.52 GHz (1.90 mm) under the low filler loading.
- A symmetric gradient honeycomb structure was constructed utilizing the high-frequency structure simulator, achieving an EAB of 14.6 GHz and a *RL*_min_ of -59.0 dB.

**Supplementary Information:**

The online version contains supplementary material available at 10.1007/s40820-024-01435-z.

## Graphical abstract

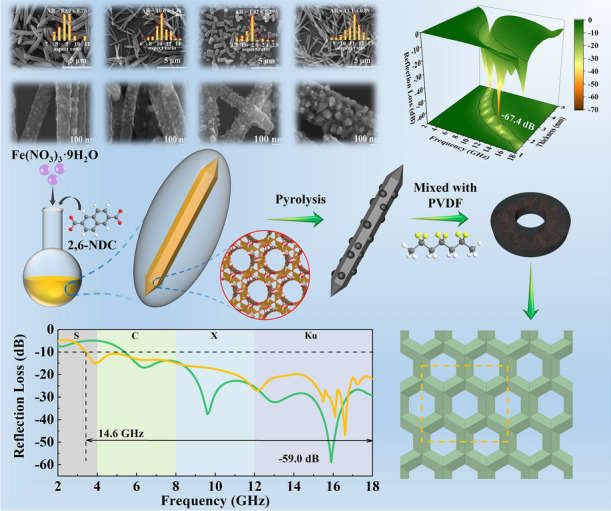

## Introduction

In the contemporary era, electromagnetic (EM) technology is undergoing continuous development and finds widespread applications in both military and civilian domains, including military radar, portable electronics, wireless communication, and more [1–3]. While EM technology provides substantial convenience, it also gives rise to electromagnetic interference and radiation, posing risks to people's wellness [4–6], and impacting the regular operation of electronic devices. To address the aforementioned challenges, the development of materials that can absorb electromagnetic waves, particularly microwaves, is an imperative. Microwave absorbing materials (MAM) can be categorized into three groups based on distinct absorption mechanisms: materials with dielectric loss, magnetic loss, and composite loss [7]. Dielectric loss materials primarily encompass carbon-based materials [8–10], MXene [11], non-magnetic metal compounds [12], and nonmetallic compounds [13]. Notably, carbon-based materials have garnered significant attention owing to their distinctive properties, including lightweight characteristics, suitability for practical engineering applications, and favorable electrical properties. Magnetic loss materials predominantly consist of magnetic metals [14] and their compounds [15]. Adequate impedance matching is a fundamental requirement for optimal microwave absorbing material (MAM) performance. Individual dielectric loss materials or magnetic loss materials alone cannot achieve effective impedance matching. Combining both types can overcome the aforementioned limitations [16].

Metal–organic frameworks (MOF) are coordination polymers comprising metal ions coordinated with organic ligands, forming intricate structures with diverse functionalities and applications. Composites comprising magnetic metals or their compounds coated with carbon can be synthesized by controlling pyrolysis conditions. These composites effectively combine dielectric losses from carbon materials and magnetic losses from either magnetic metals or their compounds, thereby enhancing impedance matching and MA performance [17]. Currently, several studies on iron-based MOF derivatives with abundant metal element reserves and low costs have been conducted. Among the iron-based MOF, the MIL-88 series comprises four subtypes: 88A (fumaric acid), 88B (terephthalic acid, 1,4-BDC), 88C (2,6-naphthalenedicarboxylic, 2,6-NDC), and 88D (4,4′-biphenyldicarboxylic, 4–4′-BPDC), distinguished by different organic ligands [18, 19]. Among them, MIL-88C is characterized by a short synthesis cycle and a large AR. According to percolation theory, the larger the AR of one-dimensional (1D) materials, the lower the percolation threshold value. This scenario is conducive to achieving optimal MA performance at low filler levels [20]. Previous studies have demonstrated that different ligands can influence the EM parameters of the corresponding derivatives, thereby impacting MA performance [21]. Wu et al. [22] utilized MIL-88A as a precursor to synthesize the absorber Fe_3_O_4_/Fe_3_C@C through pyrolysis at 600 °C. The *RL*_min_ was -52.9 dB at 11.68 GHz (3.07 mm) and a higher filler loading of 40 wt%, achieving a EAB of 4.56 GHz at 3.5 mm. Xiong et al. [23] synthesized Fe_3_O_4_@nanoporous carbon (NPC) composites by calcining Fe-MIL-88B. With a high filling amount of 30 wt%, Fe_3_O_4_@NPC exhibited a *RL*_min_ of -65.5 dB at 9.8 GHz, while exhibiting an undesirable EAB of 4.5 GHz (3 mm). All of the aforementioned absorbers exhibited high filler amounts, narrow EAB, and thick matching thicknesses. Additionally, current research has focused on investigating the impact of pyrolysis conditions on MA properties. The influence of varying preparation conditions of the precursors on the MA properties of derivatives has been scarcely investigated.

The potential for practical engineering applications of single MAM is somewhat limited by inherent constraints. Numerous materials with unique structures exhibit MA properties surpassing those of natural materials, especially EAB [24–26]. The honeycomb structure has gained prominence in current research due to its lightweight and high-strength characteristics [27, 28]. Impedance matching and attenuation capabilities are pivotal considerations in determining MA properties. Conventional through-hole honeycomb structures possess certain MA properties but do not independently isolate the two aforementioned factors. The entire honeycomb through-hole is responsible for both the entry and absorption of microwaves. Designing a structure that optimizes these two factors separately can more effectively leverage structural advantages and exhibit enhanced MA properties.

Herein, we controllably synthesized MIL-88C (Fe) as a precursor with varying AR using an oil bath under diverse conditions, followed by one-step thermal decomposition to obtain the corresponding carbon-coated iron-based composites. The influence of MIL-88C (Fe) preparation conditions on the MA properties of the derivatives was investigated, including M/O, oil bath temperature, and oil bath time. Consequently, the phases, graphitization degree, and AR of the derivatives were influenced to attain low filler loading, optimize impedance matching, and enhance MA performance. As anticipated, the composites derived from MIL-88C (Fe) fabricated under a diverse range of conditions consistently displayed outstanding MA properties, including low loading, strong absorption, broad EAB, and thin thickness. We also designed a SGHS that concentrated on optimizing impedance matching in the upper half to facilitate greater microwave entry and on enhancing attenuation capability in the lower half to enable more efficient microwave absorption. Excellent MA performance was achieved through HFSS EM simulation.

## Experiment

### Materials

All chemical reagents used in this experiment were analytically pure without further purification processes. Ferric nitrate nonahydrate (Fe(NO_3_)_3_·9H_2_O, 98.5%) and 2,6-NDC (98%) were bought from Shanghai Macklin Biochemical Co., Ltd (Shanghai, China). N,N-Dimethylformamide (DMF, 99.5%) was bought from Modern Oriental (Beijing) Technology Development Co., Ltd (Beijing, China). Methanol was purchased from Shanghai Titan Scientific Co., Ltd (Shanghai, China). Nitrogen (N_2_, high-purity) was purchased from Beijing Qianxi Jingcheng Gas Co., Ltd (Beijing, China). PVDF was obtained from Suzhou Sinero Technology Co., Ltd (Suzhou, China).

### Synthesis of MIL-88C (Fe)

MIL-88C (Fe) was synthesized using an oil bath method, a modified version of a previously reported fabrication procedure [29]. Initially, 8 mL of DMF was stirred magnetically for 10 min with 75 mg (0.18 mmol) Fe(NO_3_)_3_·9H_2_O and 4 mg (0.018 mmol) 2,6-NDC (M/O = 10:1). Subsequently, the mixture was subjected to heating in an oil bath set at 110 °C for a duration of 10 min. The obtained products underwent a triple washing procedure after natural cooling involving three cycles of rinsing with DMF and methanol, utilizing centrifugation (8000 r min^−1^, 2 min) for each step. Finally, the specimens were desiccated in a vacuum oven set at 60 °C for the entire night. The synthesized samples were designated as 88C-1 in Table 1. Simultaneously, the synthesis conditions for 88C-2, 88C-3, and 88C-4 are documented in Table 1. Notably, the quantity of the organic ligand remained constant.

**Table 1:** Naming rules for materials

MOF	Conditions		
	M/O	Oil bath temperature (°C)	Oil bath time (min)
88C-1	10:1	110	10
88C-2	10:1	100	10
88C-3	2:1	110	10
88C-4	10:1	110	30

### Fabrication of Carbon Coating Iron-Based Composites

The MIL-88C (Fe) prepared above served as the precursor and was then pyrolyzed under nitrogen at 800 °C for 2 h with a temperature rise rate of 5 °C min^−1^. The obtained specimens derived from 88C-1, 88C-2, 88C-3, and 88C-4 were named as MD_1_, MD_2_, MD_3_, and MD_4_, respectively.

### PPreparation of MOF-Derivatives/PVDF Coaxial Rings

The total mass of MOF-derivatives (MD_1_, MD_2_, MD_3_, or MD_4_) and PVDF was 120 mg. The filling amounts of MOF-derivatives were 5 wt% (6 mg), 10 wt% (12 mg), 15 wt% (18 mg), and 20 wt% (24 mg), respectively. Initially, a certain number of MOF-derivatives and PVDF were dispersed by ultrasound in 5 mL of DMF for 30 min. Then, the homogeneous dispersion was transferred to an oven to evaporate at 90 °C for 4 h to obtain a flexible black film. The flexible black film was put into a special mold and pressed at 220 °C and 5 MPa for 15 min, then naturally cooled to room temperature to obtain the coaxial ring (Φ_out_ = 7.00 mm and Φ_in_ = 3.04 mm).

### Material Characterization

The tests of crystal structure and phase composition of materials were conducted utilizing a Rigaku Smartlab SE X-ray diffraction (XRD) instrument employing Cu Kα radiation with a scanning rate of 5° min^−1^. The surface features and internal constitution of the specimens were investigated via scanning electron microscopy (SEM, ZEISS Sigma 300) and transmission electron microscopy (TEM, FEI TECNAI F20). The elemental composition and spatial distribution were characterized via energy-dispersive X-ray spectrometry (EDS, Ultim Max 100). Raman spectra were documented using a high-resolution confocal microscopy laser Raman instrument (WITec alpha300R) with a 532 nm laser source. The elemental electronic structure and valence state of the materials surface were revealed via X-ray photoelectron spectroscopy (XPS, Thermo Scientific K-Alpha) employing Al Kα radiation. The magnetic hysteresis loops were tested by vibrating sample magnetometer (VSM, Lake Shore 7404). Thermogravimetry (TG, Netzsch STA 449 F5 Jupiter) analysis was carried out under N_2_ atmosphere with a heating rate of 10 °C min^−1^. Fourier transform infrared (FT-IR) spectra were acquired using an IRAffinity-1 spectrometer with spectral grade KBr sheets. The EM parameters of the coaxial rings were measured with a coaxial approach using a vector network analyzer (VNA Agilent TE5071C).

## Results and Discussion

### Characterizations of the As-Synthesized Materials

**Fig. 1:**
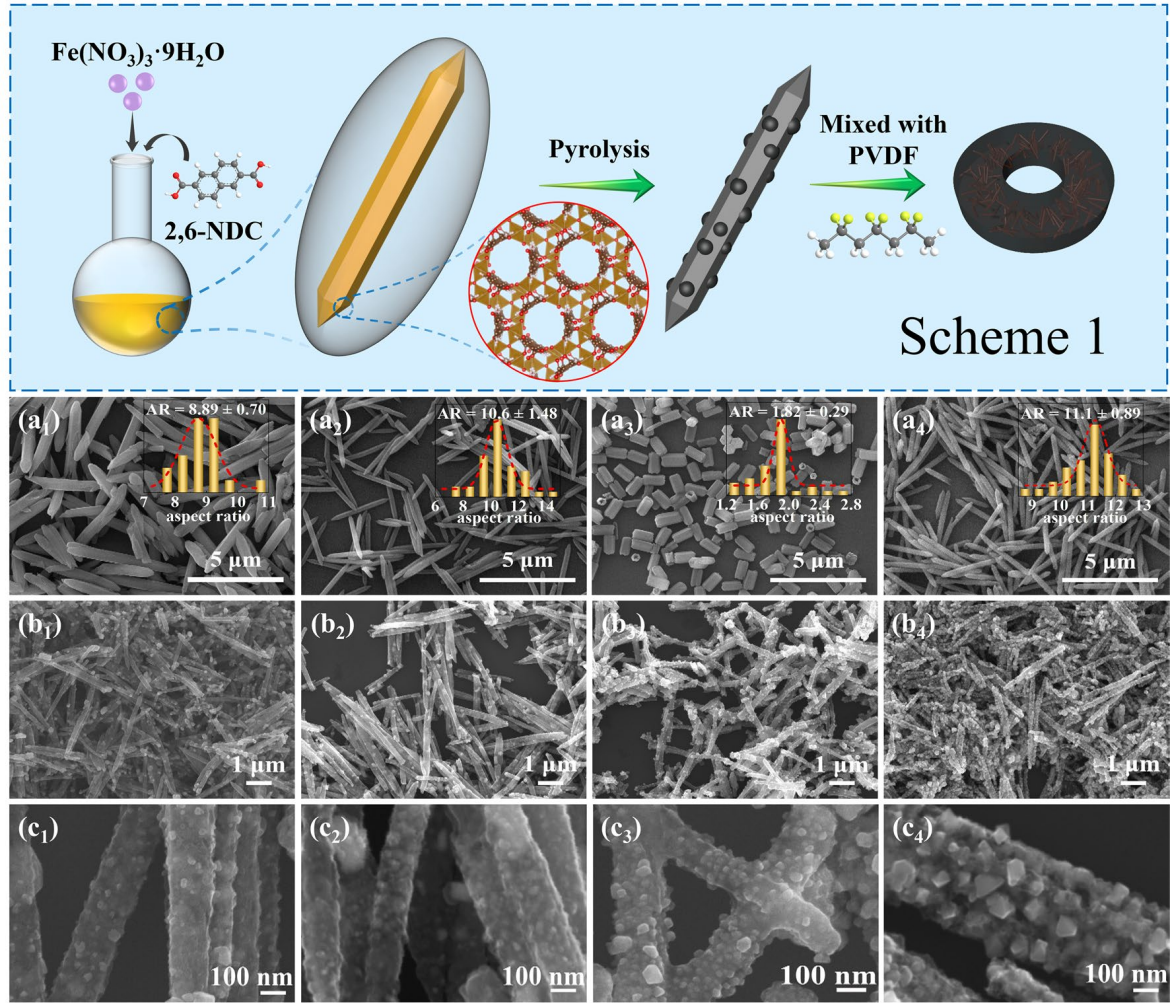
(Scheme 1) Forming process of carbon-coated iron-based composites and absorbers; SEM images and AR normal distribution of precursors: **a**_**1**_ 88C-1, **a**_**2**_ 88C-2, **a**_**3**_ 88C-3, and **a**_**4**_ 88C-4; Corresponding derivatives and their local enlargement: **b**_**1**_**, c**_**1**_ MD_1_, **b**_**2**_**, c**_**2**_ MD_2_, **b**_**3**_**, c**_**3**_ MD_3_, and **b**_**4**_**, c**_**4**_ MD_4_

The preparation process of the derivatives from MIL-88C (Fe) and the absorbers mixed with PVDF is depicted in Scheme 1. The MIL-88C (Fe) has a three-dimensional (3D) hexagonal structure, in which trimeric units of Fe_3_O are linked by NDC^2–^ to form a periodic 3D structure of [Fe_3_O(NDC)_3_(H_2_O)_2_(NO_3_)]_*n*_ [29, 30]. Anisotropic growth leads to a hexagonal rod shape (Fig. 1a). Interestingly, the M/O, oil bath temperatures, and oil bath times were able to change AR. Specifically, increasing the M/O, lowering the oil bath temperature, or extending the oil bath time can raise the AR of MIL-88C (Fe). Iron ions (Fe^3+^) play a significant role in directional growth, and the larger their amount, the longer the nanorods grow. The temperature of the oil bath affects the nucleation rate. The lower the temperature, the fewer the crystal nuclei, and accordingly, the longer the nanorods will be. The prolongation of reaction time leads to a more complete growth of the crystal along the axial direction. In addition, the sample surfaces became rough, and some small crystals were observed on their surfaces when the oil bath time was long enough. This phenomenon reveals that dissolution–recrystallization may occur on the material surfaces. The pyrolysis process consists of two stages: carbonization and graphitization. For pure organic materials, the graphitization process requires high temperatures above 1000 °C. If metal components are used as a catalyst, the temperature required for graphitization can be reduced to below 1000 °C [31]. Therefore, in this work, iron and metal compounds containing Fe, generated during pyrolysis, play catalytic roles in the graphitization process. After pyrolysis, the derivatives shrank in size due to the decomposition of organic ligands. And their surfaces became rough with a large number of nanoparticles of variable sizes evenly distributed on surfaces, but they still retained the basic shape of the precursors (Fig. 1b/1c). In the case of MD_3_, for example, the results of EDS indicated the content of O was higher than it should be in Fe_3_O_4_ (Fig. S1). It demonstrates that there are other forms of O present. Figure S2 illustrates the FT-IR results of MD_3_, with peaks observed at 1634 and 1400 cm^−1^ corresponding to the symmetric and asymmetric stretching modes, respectively, of the carboxyl groups in 2,6-NDC [32]. Furthermore, the fact that the derivative retained the basic shape of the precursor also indicated that the organic ligand had not completely collapsed. Both of the above results suggest that the excess O is present in organic ligands.

**Fig. 2:**
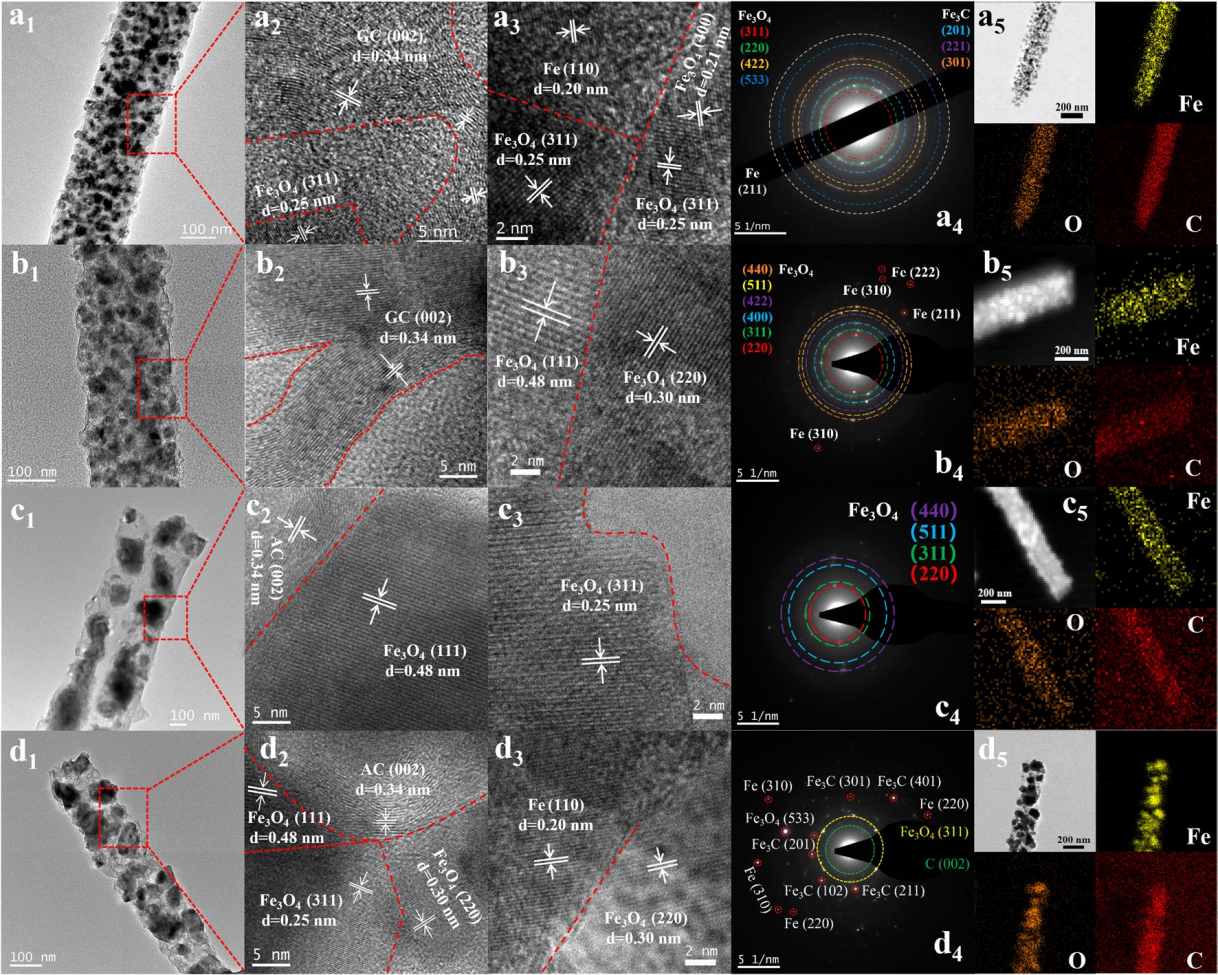
**1** TEM images, **2 ~ 3** HRTEM images, **4** SAED patterns, and **5** element mapping for Fe, O, and C of **a** MD_1_, **b** MD_2_, **c** MD_3_, and **d** MD_4_

TEM and HRTEM were used to further obtain the component and structural information of materials (Fig. 2). For MD_1_, MD_2_, MD_3_, and MD_4_, the TEM results all showed that the iron-based components were encapsulated in carbon. The iron-based phases of MD_1_ and MD_2_ exhibited small and dense structures (Fig. 2a_1_/b_1_), whereas MD_3_ and MD_4_ displayed bulky and sparse characteristics (Fig. 2c_1_/d_1_). The HRTEM results clearly revealed lattice stripes representing different components or various crystal faces of a specific component (Fig. 2a_2_ ~ d_2_/a_3_ ~ d_3_). The distinct lattice stripes of 0.48, 0.30, and 0.25 nm corresponded to the (111), (220), and (311) planes of Fe_3_O_4_, respectively (Fig. 2b_3_, c_2~3_, d_2~3_). The lattice fringes of 0.20 nm were attributed to the (110) plane of Fe (Fig. 2d_3_). The curved lattice fringes of 0.34 nm in Fig. 2a_2_ ~ d_2_ belonged to the (002) plane of graphitic carbon (GC), where the lattice fringes in Fig. 2a_2_/b_2_ were more continuous and multilayered. The diffraction circles and spots observed in the selected area electron diffraction (SAED) patterns further demonstrated the existence of the corresponding phases (Fig. 2a_4_ ~ d_4_), proving the polycrystalline structure. The elemental mapping results demonstrated the existence and even dispersion of Fe, O, and C elements (Fig. 2a_5_ ~ d_5_).

**Fig. 3:**
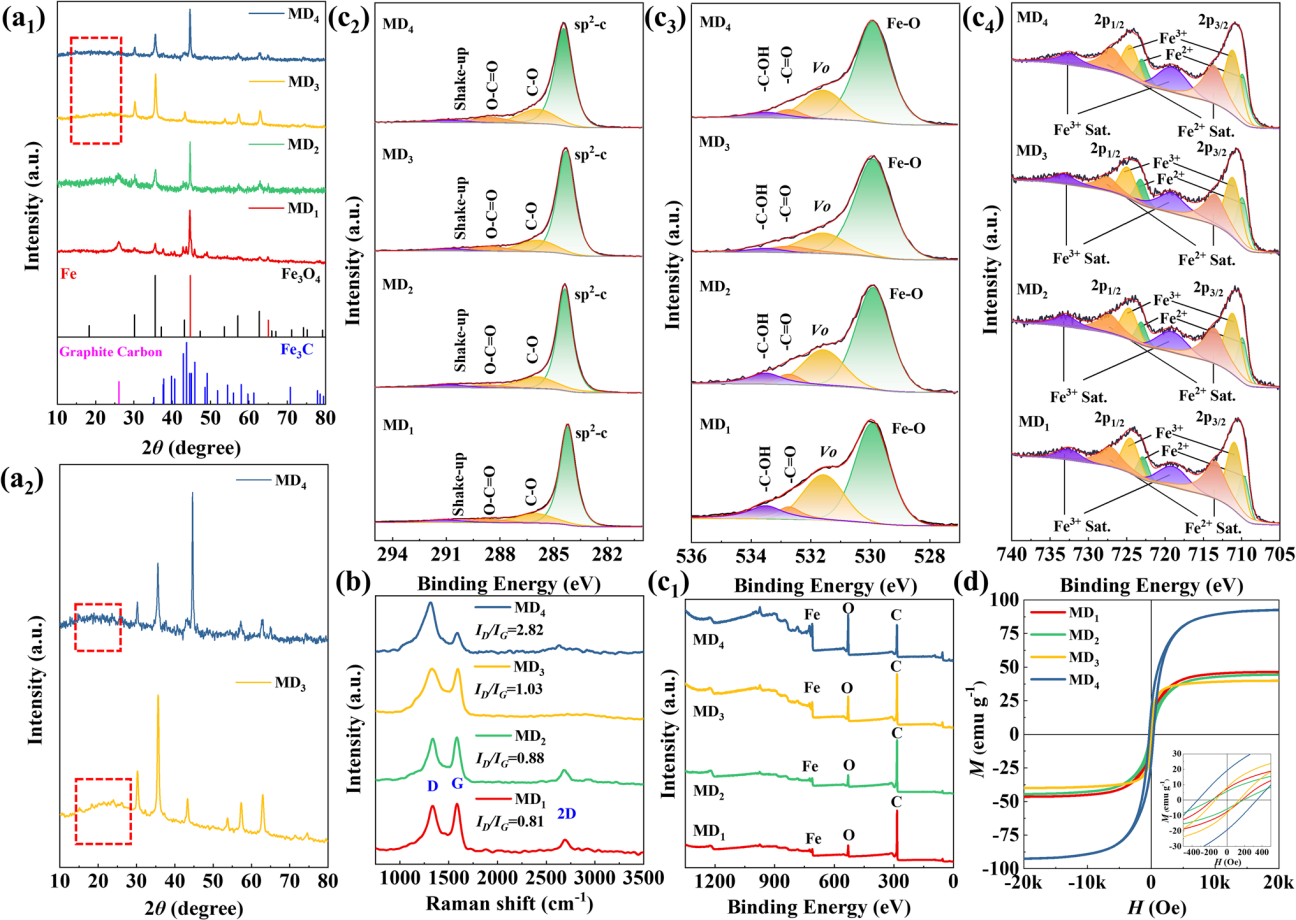
**a**_**1**_ XRD images, **a**_**2**_ XRD localized enlargement, **b** Raman spectra, XPS spectra: **c**_**1**_ survey spectrum, **c**_**2**_ C 1*s*, **c**_**3**_ O 1*s*, and **c**_**4**_ Fe 2*p*, and **d** hysteresis loops of MD_1_, MD_2_, MD_3_, and MD_4_

The XRD result (Fig. S3) indicated the successful synthesis of precursor MIL-88C (Fe). As shown in Fig. 3a_1_, Fe-containing phases in the derivatives included Fe (JCPDS: 06–0696), Fe_3_O_4_ (JCPDS: 88–0315), or Fe_3_C (JCPDS: 34–0001). For the carbon component, MD_1_ and MD_2_ were dominated by GC (JCPDS: 34–0567) (Fig. 3a_1_). The broad peaks of MD_3_ and MD_4_ at about 20~30° represented the (002) plane of amorphous carbon (AC) (Fig. 3a_2_) [33]. The specific phases of the derivatives were as follows: MD_1_ and MD_4_ (Fe/Fe_3_O_4_/Fe_3_C@C), MD_2_ (Fe/Fe_3_O_4_@C), and MD_3_ (Fe_3_O_4_@C), in good agreement with HRTEM and SAED results. Notably, Fe phases are present in all the composites except MD_3_. This occurrence may be attributed to the high M/O conditions, where the O content of the organic ligand is not sufficient to react with all Fe^3+^ ions to form Fe_3_O_4_. Consequently, the surplus Fe^3+^ ions are reduced to Fe phases.

Raman spectroscopy, an effective bulk characterization method, is used for analyzing the carbonaceous materials. The peaks at the 1350 and 1580 Raman shifts in Fig. 3b represent the D and G peaks of the carbonaceous materials. The D peak serves as an indicator of imperfections within the carbonaceous materials, here including *sp*^3^-C in amorphous carbon and flaws in the *sp*^2^-hybridized mesh. The G peak indicates perfect graphite consisting of *sp*^2^-hybridized carbon [34, 35]. The *I*_D_*/I*_G_ intensity ratio is frequently employed to depict both the level of graphitization and the presence of defects within carbon materials. Figure 3b shows the Raman spectra of the derivatives. The *I*_D_*/I*_G_ results for MD_1_, MD_2_, MD_3_, and MD_4_ were 0.81, 0.88, 1.03, and 2.82, respectively, indicating a decreasing order in the degree of graphitization. The aforementioned findings are also in alignment with the carbon diffraction peaks in the XRD results. The degree of graphitization was related to the type and size of the Fe-containing phases used as catalysts. Specially, the MD_2_ had iron-based components with the same small sizes as MD_1_ but lacked the Fe_3_C phase, which made it have a slightly lower graphitization degree than MD_1_. For MD_3_, it only had Fe_3_O_4_ with a larger size and therefore had an elevated value of *I*_D_*/I*_G_ as well as a lower level of graphitization. Although MD_4_ had the same iron-based components as MD_1_, the larger size of the iron-based phases left less room for the carbon component and therefore also had a lower degree of graphitization. Moreover, the broad 2D peaks near 2700 cm^−1^ represent the presence of several layers of graphite, and the narrow 2D peaks represent multiple layers of graphite [36]. The conclusions drawn from the 2D peaks were in accordance with the HRTEM results. The above results indicate that the content of Fe^3+^, the oil bath temperature, and the oil bath time affect the phases and degree of graphitization of the derivatives.

The XPS results are presented in Fig. 3c. The survey spectra (Fig. 3c_1_) showed that the composites contained C, O, and Fe elements. The intensity of the C peaks indicated that MD_1_ and MD_2_ had higher and similar carbon content, whereas MD_3_ and MD_4_ exhibited sequentially lower carbon content. The peaks at 284.3 eV represented *sp*^2^-C, which demonstrated the material surface to be mainly graphite (Fig. 3c_2_). Meanwhile, the peaks representing carboxyl groups from the undecomposed organic ligand 2,6-DNC were also present. In the narrow spectra of the O element (Fig. 3c_3_), the two peaks with lower binding energies were attributed to the lattice oxygen (529.9 eV) and vacancy oxygen (*Vo*) in Fe_3_O_4_ (531.6 eV) [37]. The two remaining peaks at 532.7 eV and 533.5 eV denoted the oxygen in the carboxyl group, further demonstrating the existence of the undecomposed organic ligand 2,6-DNC. As shown in Fig. 3c_4_, the Fe element mainly consisted of two valence states of “ + 2” and “ + 3,” which indicated that the material surface was mainly Fe_3_O_4_, while the Fe and Fe_3_C were mainly present in the bulk phases.

The magnetic properties of the composites are reflected by the saturation magnetization (*M*_s_) and coercivity (*H*_c_) from the VSM results (Fig. 3d). In particular, MD_4_ presented a ferromagnetic hysteresis loop with the maximum *M*_s_ of 92.5 emu g^−1^ and *H*_c_ of 331.6 Oe, which is due to the fact that it contains multifarious and bulky ferromagnetic components and low carbon content. MD_3_ displayed the minimal *M*_s_ of 39.9 emu g^−1^ and *H*_c_ of 131.5 Oe. This is attributed to the reason that it has only a single magnetic component Fe_3_O_4_ and high carbon content. The values of *M*_s_ and *H*_c_ for MD_1_ (46.4 emu g^−1^, 161.2 Oe) and MD_2_ (44.5 emu g^−1^, 181.2 Oe) are almost equal. The *M*_s_ of MD_2_ is slightly lower than that of MD_1_ due to the absence of Fe_3_C in MD_2_ compared to MD_1_. Although MD_1_ and MD_4_ have the same variety of ferromagnetic compositions, MD_1_ has a higher carbon content than MD_4_. Therefore, the *M*_s_ and *H*_c_ of MD_1_ are considerably lower than those of MD_4_.

To determine the suitable temperature for obtaining carbon-coated iron-based composites, the TG curve of 88C-3 under N_2_ conditions was examined (Fig. S4). The mass loss was 4.4 wt% in stage I, corresponding to the loss of guest H_2_O molecules. The mass loss of 7.6 wt% in stage II was attributed to the removal of coordinated H_2_O and DMF molecules. With the increase in temperature, the large mass loss of 26.7 wt% in stage III corresponded to the decomposition of organic ligands. The mass loss of 12.7 wt% in the last stage IV indicated complete decomposition of organic ligands [38], culminating at ~ 800 °C with a total weight loss of 51.4 wt%. Consequently, derivatives with adequate pyrolysis can be obtained at 800 °C.

### MA Performance of Absorbers Derived From MIL.88C (Fe)

The values of *RL* and EAB are major indicators to assess the MA properties. *RL* values can be counted using the subsequent two equations [39]:

1 $$RL = 20\log \left| {\frac{{Z_{{{\text{in}}}} - Z_{0} }}{{Z_{{{\text{in}}}} + Z_{0} }}} \right|$$

2 $$Z_{{{\text{in}}}} = Z_{0} \sqrt {\frac{{\mu_{{\text{r}}} }}{{\varepsilon_{{\text{r}}} }}} \tanh \left[ {j\left( {\frac{2f\pi d}{c}} \right)\sqrt {\mu_{{\text{r}}} \varepsilon_{{\text{r}}} } } \right]$$

where the normalized input impedance of the absorbers and free space is represented by *Z*_in_ and *Z*_0_, respectively. *ε*_r_ and *μ*_r_ denote the relative complex permittivity (*ε*_r_ = *ε*′ − *jε*″) and complex permeability (*μ*_r_ = *μ*′ − j*μ*″). *f* represents the incident EM wave frequency, *d* denotes the thickness of the absorbers, and *c* signifies the speed of light propagation in vacuum. *RL* < -10 dB means the absorber possesses MA properties, and EAB designates the frequency region wherein *RL* < -10 dB. To fully exploit the MA performance of the absorbers, we chose PVDF instead of wax as the matrix. The effect of PVDF and wax as substrates on the MA properties was compared using MD_3_ as an example (Fig. S5). As shown in Fig. S5a_1/2_, the *ε*′ and *ε*″ of MD_3_/PVDF are significantly higher than those of MD_3_/wax for the same filling amount (10 wt%), consistent with previous studies [40]. The notable increase in the dielectric constant is attributed to dipole polarization resulting from the difference in electronegativity between F/H atoms and C atoms, as well as interfacial polarization occurring between PVDF and MD_3_ [41, 42]. The value of *μ*′ and *μ*″ has not changed significantly (Fig. S5b_1/2_). Therefore, choosing PVDF as the substrate can significantly enhance the MA properties (Fig. S5c_1/2_). Additionally, PVDF can be utilized to construct flexible MAM characterized by corrosion resistance and excellent mechanical properties. It also demonstrates process feasibility and practicality in engineering applications compared to wax substrates.

**Fig. 4:**
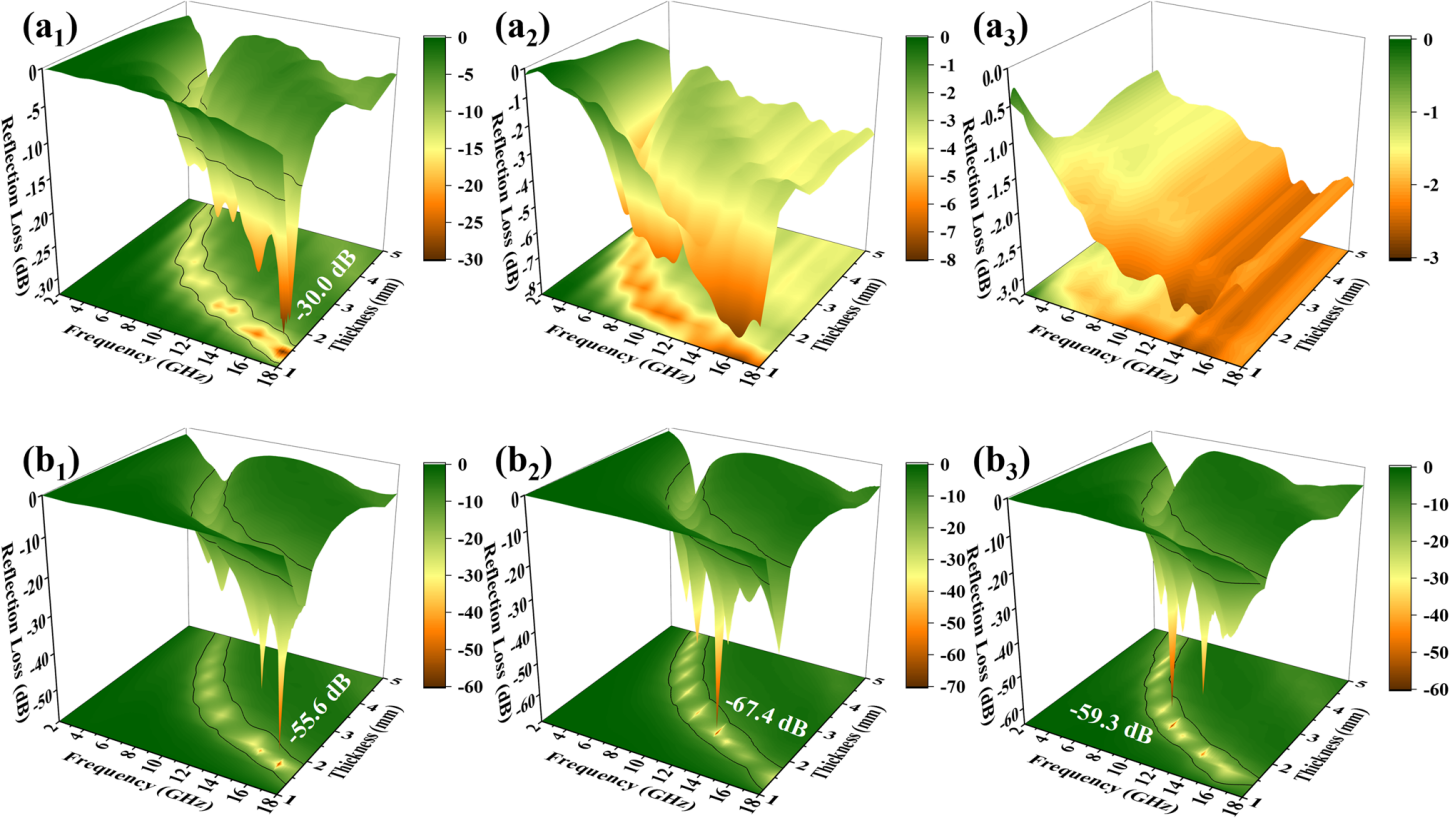
3D RL curves of MD_1_/PVDF at filler loading of **a**_**1**_ 5 wt%, **a**_**2**_ 10 wt%, and **a**_**3**_ 20 wt%; **b**_**1**_ MD_2_/PVDF at filler loading of 5 wt%, **b**_**2**_ MD_3_/PVDF at filler loading of 10 wt%, and **b**_**3**_ MD_4_/PVDF at filler loading of 20 wt%

As shown in Fig. 4a_1_ and S6a_1_, MD_1_/PVDF (5 wt%) had a minimum *RL* of -30.0 dB at 17.6 GHz (1.4 mm) and the EAB was 4.8 GHz (1.61 mm). As the filling amount increased, the MA properties became progressively worse (Fig. 4a_2/3_ and Fig. S6a_2/4_). In comparison, the optimal *RL* of MD_2_/PVDF (5 wt%) rose to -55.6 dB at 16.64 GHz (1.72 mm), and the EAB broadened to 5.52 GHz (1.90 mm) (Fig. 4b_1_ and Fig. S6b_1_). Compared to MD_1_/PVDF at the same filling amounts, MD_3_/PVDF and MD_4_/PVDF achieved a leap from none to excellent MA properties. In Fig. 4b_2_ and Fig. S6c_2_, the *RL*_min_ of MD_3_/PVDF (10 wt%) was -67.4 dB at 12.56 GHz (2.13 mm), and the EAB was 4.96 GHz (1.78 mm). In Fig. 4b_3_ and Fig. S6d_4_, the *RL*_min_ of MD_4_/PVDF (20 wt%) was -59.3 dB at 10.4 GHz (2.17 mm), and the EAB was 4.88 GHz (1.56 mm). The filler loadings of absorbers have a significant effect on the MA performance. The MA properties of MD_1_/PVDF, MD_2_/PVDF, MD_3_/PVDF, and MD_4_/PVDF at various filler loadings are displayed in Fig. S6 and Fig. S7.

Notably, the graphitization degree and AR together influence the optimum filler loading, with the degree of graphitization being the main factor. The greater the level of graphitization, the more readily a conductive network. MD_1_ had the highest degree of graphitization and a large AR, resulting in an optimal filler amount as low as 5 wt%. MD_2_ was slightly less graphitized than MD_1_ but had a larger AR than MD_1_, so the optimum filler loading could also be as low as 5 wt%. Compared to MD_1_, MD_3_ had a low degree of graphitization and a small AR; thus, it had an optimal filler amount of 10 wt%. Although MD_4_ had the largest AR, the degree of graphitization was the lowest, therefore the optimum filler loading was 20 wt%.

**Fig. 5:**
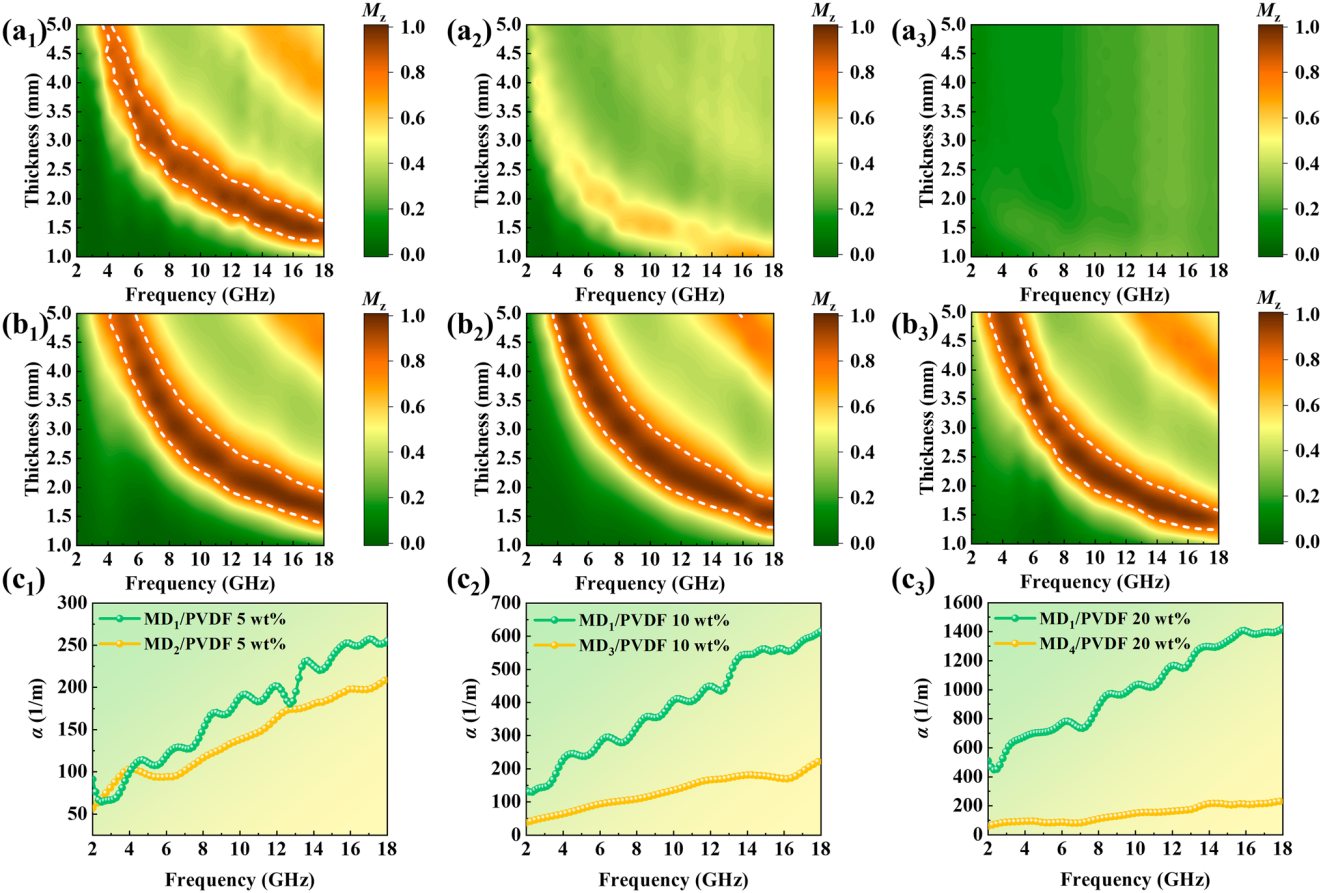
2D contour map of the *M*_z_ value of MD_1_/PVDF at filler loading of **a**_**1**_ 5 wt%, **a**_**2**_ 10 wt%, and **a**_**3**_ 20 wt%; **b**_**1**_ MD_2_/PVDF at filler loading of 5 wt%, **b**_**2**_ MD_3_/PVDF at filler loading of 10 wt%, and **b**_**3**_ MD_4_/PVDF at filler loading of 20 wt%; **c**_**1**_** ~ c**_**3**_ Contrast values for attenuation constants *α*

Generally speaking, two essential factors dictate the MA properties. The primary consideration is impedance matching coefficient (*M*_z_). For samples with finite thickness, utilizing *M*_z_ to evaluate impedance matching characteristics is more reasonable [43], and value near 1.0 indicates a well-matched impedance, with a large number of EM waves able to be introduced into materials. An assessment of the impedance matching capability of absorbers is conducted within the range of 0.8 to 1.0 [34]. Besides good impedance matching, MA performance is also influenced by the attenuation constant (*α*). The value of *M*_z_ and α were determined by the subsequent equations [34, 44]:

3 $$M_{\text{z}}=\frac{2Z_{\text{in}}^{'}}{\vert Z_{\text{in}}\vert^{2}+1}$$

4 $$\alpha = \frac{\sqrt 2 \pi f}{c}\sqrt {(\mu^{\prime \prime } \varepsilon^{\prime \prime } - \mu^{\prime } \varepsilon^{\prime } ) + \sqrt {(\mu^{\prime \prime } \varepsilon^{\prime \prime } - \mu^{\prime } \varepsilon^{\prime } )^{2} + (\mu^{\prime } \varepsilon^{\prime \prime } + \mu^{\prime \prime } \varepsilon^{\prime } )^{2} } }$$

where the *Z*_in_^′^ is the real input impedance, where the parameters *ε′, μ′*, *ε′′,* and *μ''* are real and imaginary parts of relative complex permittivity and permeability, respectively. As shown in Fig. 5a_1_/b_1_, MD_2_/PVDF (5 wt%) possessed a greater impedance matching area delineated with the white dotted lines and more *Z* values close to 1 than MD_1_/PVDF (5 wt%). The values of *α* for both are close to each other, indicating similar attenuation capacities (Fig. 5c_1_). As a result, MD_2_/PVDF (5 wt%) had a stronger RL and a wider EAB. In Fig. 5a_2_/a_3_, MD_1_/PVDF (10 wt%) and MD_1_/PVDF (20 wt%) had poor impedance matching. This is due to the high graphitization degree, which makes MD_1_/PVDF easier to form conductive networks, resulting in higher dielectric constants within high filler loadings (Fig. S8). Notably, for MD_1_/PVDF within a filler loading of 20 wt%, the *ε′′* is greater than *ε′*, which indicates the formation of a conductive network resembling a conductor at high filler levels, leading to skin effects [45]. Due to the impedance mismatch, even though MD_1_/PVDF (10 wt%) and MD_1_/PVDF (20 wt%) had higher loss capacities (Fig. 5c_2_/c_3_), they could not exhibit MA properties. In contrast, both MD_3_/PVDF (10 wt%) and MD_4_/PVDF (20 wt%) had excellent impedance matching (Fig. 5b_2_/b_3_), combined with suitable dissipation capacities, and therefore exhibited excellent MA performance.

EM parameters were analyzed in depth for the purpose of further exploring the MA mechanism of the three excellent absorbers. The real parts (*ε*′ and *μ*′) signify the storage capability of electrical and magnetic energy, while the imaginary parts (*ε″* and *μ″*) indicate the ability to dissipate EM energy. The *ε*′ values in Fig. S9a_1_ exhibit an overall downward trend from 13 to 7, 8, or 10 approximately with increasing frequency, exhibiting the dispersion effect. The *ε″-f* graphs of the three absorbers were observed in Fig. S9a_2_, and the curves all exhibited an overall decreasing trend from 7.6 to 3.6, 5.2 to 3.3, and 5.7 to 2.9, respectively. This represents the conductivity loss and small resonance peaks represent the polarization loss [46]. The MD_4_/PVDF had the highest number of resonant peaks, the MD_2_/PVDF the second most, and the MD_3_/PVDF the least. The dielectric loss tangent (tan*δ*_*ε*_ = *ε′′*/*ε′*) was computed to assess the ability of absorbers to lose electrical energy, and the corresponding curves showed the same trend as *ε″* (Fig. S9a_3_)*.* Figure S9b_1/2_ display the *μ′-f* and *μ″-f* graphs of the three absorbers, and the presence of resonance peaks is observed, indicating that the absorbers have magnetic loss capability to some extent. Some negative *μ″* values of the MD_2_/PVDF may result from the induced alternating magnetic field generated by the motion of free electrons within the alternating electric field, leading to the radiation of magnetic energy from the conductive networks [47]. The values of the magnetic loss tangent (tan*δ*_*μ*_ = *μ″*/*μ*) are also computed to assess the ability to lose magnetic energy (Fig. S9b_3_). By comparison, the value of tan*δ*_*ε*_ is higher than tan*δ*_*μ*_, demonstrating that the contribution of dielectric loss to the MA performance of absorbers predominates over magnetic loss.

Types of microwave loss can be classified from both component and micro/macroscopic geometric structure perspectives. The component level includes dielectric loss caused by dielectric materials and magnetic loss from magnetic materials. Dielectric loss comprises conduction loss and polarization relaxation within the range of 2 ~ 18 GHz. The Debye relaxation theory elucidates the mechanism underlying dielectric loss, and it can be summarized the correlation for *ε′* and *ε′′* as [48]:

5 $$\left( {\varepsilon^{\prime } - \frac{{\varepsilon_{{\text{s}}} + \varepsilon_{\infty } }}{2}} \right)^{2} + \left( {\varepsilon^{\prime \prime } } \right)^{2} = \left( {\frac{{\varepsilon_{{\text{s}}} + \varepsilon_{\infty } }}{2}} \right)^{2}$$

where *ε*_s_ stands for the static dielectric constant as the frequency approaches 0, while *ε*_*∞*_ denotes the permittivity in the limit as frequency approaches infinity. The *ε′*-*ε′′* curve of the three absorbers exhibited a shape accompanied by several Cole–Cole semicircles in the high frequency range and a straight line in the low frequency range in Fig. S9c_1~3_. This indicates that the dielectric loss included the contributions of polarization and conduction losses. The semicircles depict the process of relaxation polarization, encompassing dipole polarization, defect-induced polarization, and interface polarization. The dipole polarization was caused by the variance in electronegativity between F/H atoms and C atoms in PVDF and dangling bonds on the surface of Fe_3_O_4_ [49]. The defect-induced polarization was generated by carbon defects [50], grain boundaries [51], and *Vo* in Fe_3_O_4_ [52]. The interface polarization was formed by various heterogeneous interfaces with different electronegativity, including GC, AC [53], PVDF, and iron-based components. The MD_4_/PVDF had the largest number of semicircles due to the most iron components (Fe, Fe_3_O_4_, and Fe_3_C), and these iron components produced more interfacial polarization. Additionally, the number of semicircles observed was in accordance with the regularity of resonance peak occurrences in the *ε′′-f* curves. The straight lines demonstrate the conductive loss attributed to GC. Generally, the magnetic loss within the 2 ~ 18 GHz range includes eddy current loss, natural resonance, and exchange resonance. The analysis of magnetic loss type involves the calculation of the value of *C*_0_ using the following equation [54]:

6 $$C_{0} = \mu^{\prime \prime } (\mu^{\prime } )^{ - 2} f^{ - 1}$$

In Fig. S10, the three absorbers all exhibited a clear fluctuation trend between 2 ~ 8 GHz and a constant trend within the band of 8 ~ 18 GHz. This phenomenon proves that the magnetic loss was caused by natural resonance (2 ~ 8 GHz) and eddy current loss (8 ~ 18 GHz). As MD_2_/PVDF and MD_4_/PVDF contained more magnetic components, both fluctuated more significantly between 2 ~ 8 GHz, indicating more significant magnetic resonance losses occurred.

Besides the component perspective, the types of microwave loss in terms of micro/macroscopic geometric structure, including dissipation of the reflected EM wave and multiple scattering, also play a key role. The dissipation of the reflected EM wave can be analyzed based on λ/4 theory via the subsequent equation [55]:

7 $$t_{{\text{m}}} = \frac{n\lambda }{4} = \frac{nc}{{4f_{{\text{m}}} \sqrt {\left| {\varepsilon_{{\text{r}}} } \right|\left| {\mu_{{\text{r}}} } \right|} }}\left( {n = 1,3,5 \ldots } \right)$$

where *t*_m_ represents the matching thickness, and *f*_m_ represents for the frequency corresponding to the optimal *RL* value under the matching thickness. *λ* and *c* are the corresponding microwave lengths and the velocity of light in vacuo. *|ε*_r_*|* and |*μ*_r_| denote the moduli of the relative complex permittivity and permeability. The *λ*/4 model leads to a different phase of π between reflection waves from the front and back of absorbers, which results in a reflection wave extinction effect and promotes MA performance [55]. The coordinate points corresponding to the actual situation of the three absorbers were compliant to the *f*_m_ − *t*_m_ curve within the error range (Fig. S11) [43]. It demonstrates that optimum *RL* versus matching thickness is in accordance with a specific rule and the optimal *RL* values are obtained at the most matched thicknesses. Meanwhile, the *M*_z_ values at optimum thicknesses are extremely close to 1.0. Furthermore, randomly distributed nanorods tend to scatter EM waves multiple times, extending the propagation path and facilitating the conversion of EM energy into heat.

### MA Mechanisms of Derivatives From MIL.88C (Fe)

**Fig. 6:**
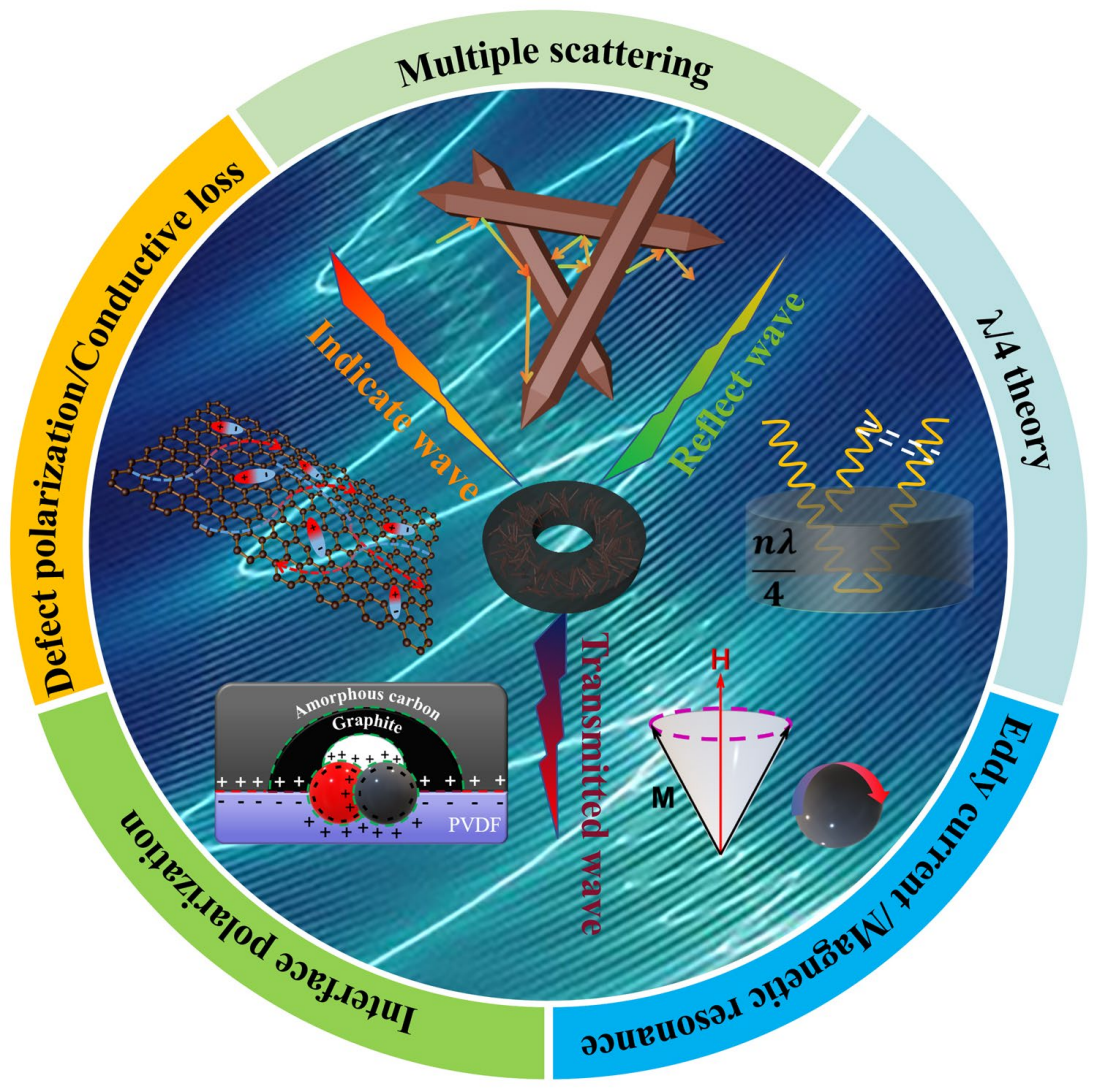
Schematic illustration of MA mechanisms for the derivatives of MIL-88C (Fe)

The potential mechanisms of MA for the absorbers are illustrated in Fig. 6. The combination of moderate dielectric constants and certain permeabilities facilitated optimal impedance matching and obtained suitable attenuation constants. The random distribution of graphite-containing derivatives constituted a 3D conductive network that promoted electron migration and hopping, thereby exhibiting conductive loss properties. Defects such as carbon defects, vacancies, and grain boundaries in the composites induced and trapped electrons, resulting in polarization induced by defects. Dipole polarizations were generated by inherent dipoles in PVDF and dangling bonds on Fe_3_O_4_ surfaces. More interfacial polarizations were also created among multiple phases. The magnetic components Fe, Fe_3_O_4_, and Fe_3_C converted EM energy into thermal energy through magnetic resonance and eddy currents. All the mechanisms discussed above were examined at the component level. Furthermore, micro/macroscopic geometric structural aspects such as multiple scattering and λ/4 theory were also significant in MA.

### Comparison of MA Performance

Eventually, a specific reflection loss (SRL) value is introduced to properly assess the MA capability considering both filler loading and thickness. It can be calculated as follows [56]:

8 $${\text{SRL}} = \frac{{{\text{RL}}}}{{{\text{Filling}} \;{\text{loading}} \times {\text{Thickness}}}}$$

**Fig. 7:**
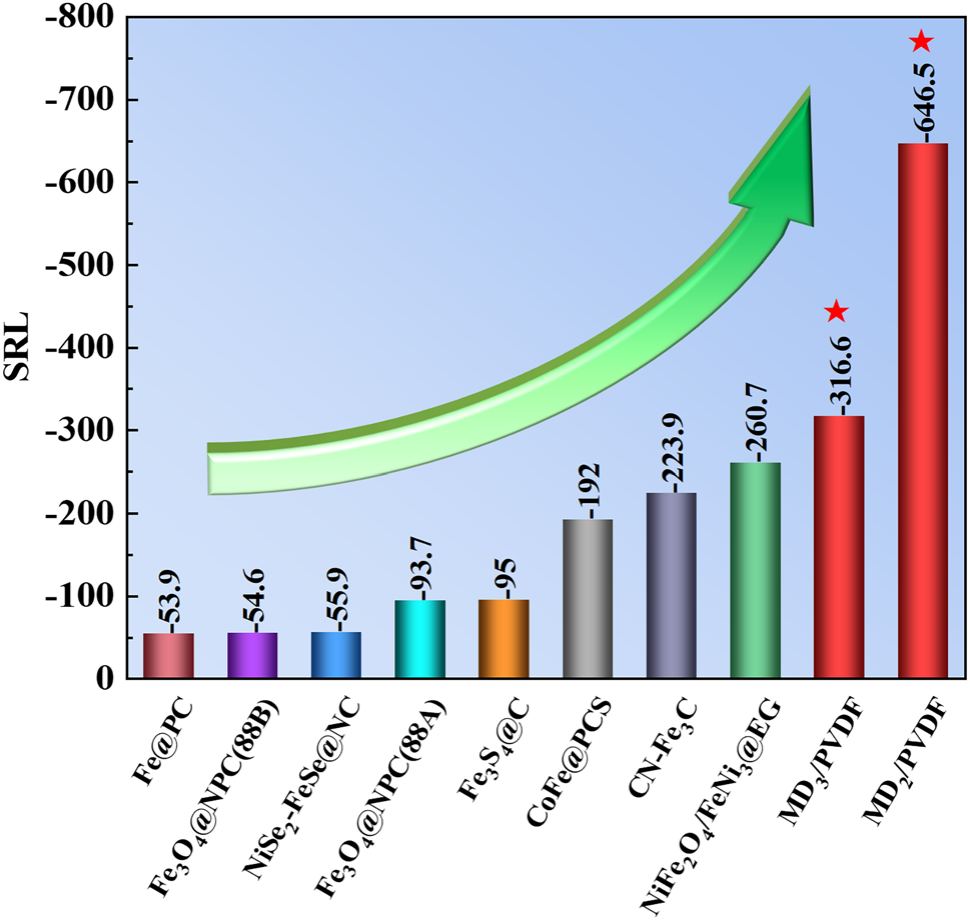
Comparison of SRL of MD_2_/PVDF and MD_3_/PVDF with the current advanced absorbers

We compared the SRL values of the MD_2_/PVDF and MD_3_/PVDF with those of recent advanced absorbers, as shown in Fig. 7. The specific performance data of the above absorbers are listed in Table S1. Surprisingly, our work exhibited minimal SRL values compared to other absorbers, indicating stronger reflection loss, thinner thickness, and lower filler loading.

### EM Simulation Results of the SGHS

**Fig. 8:**
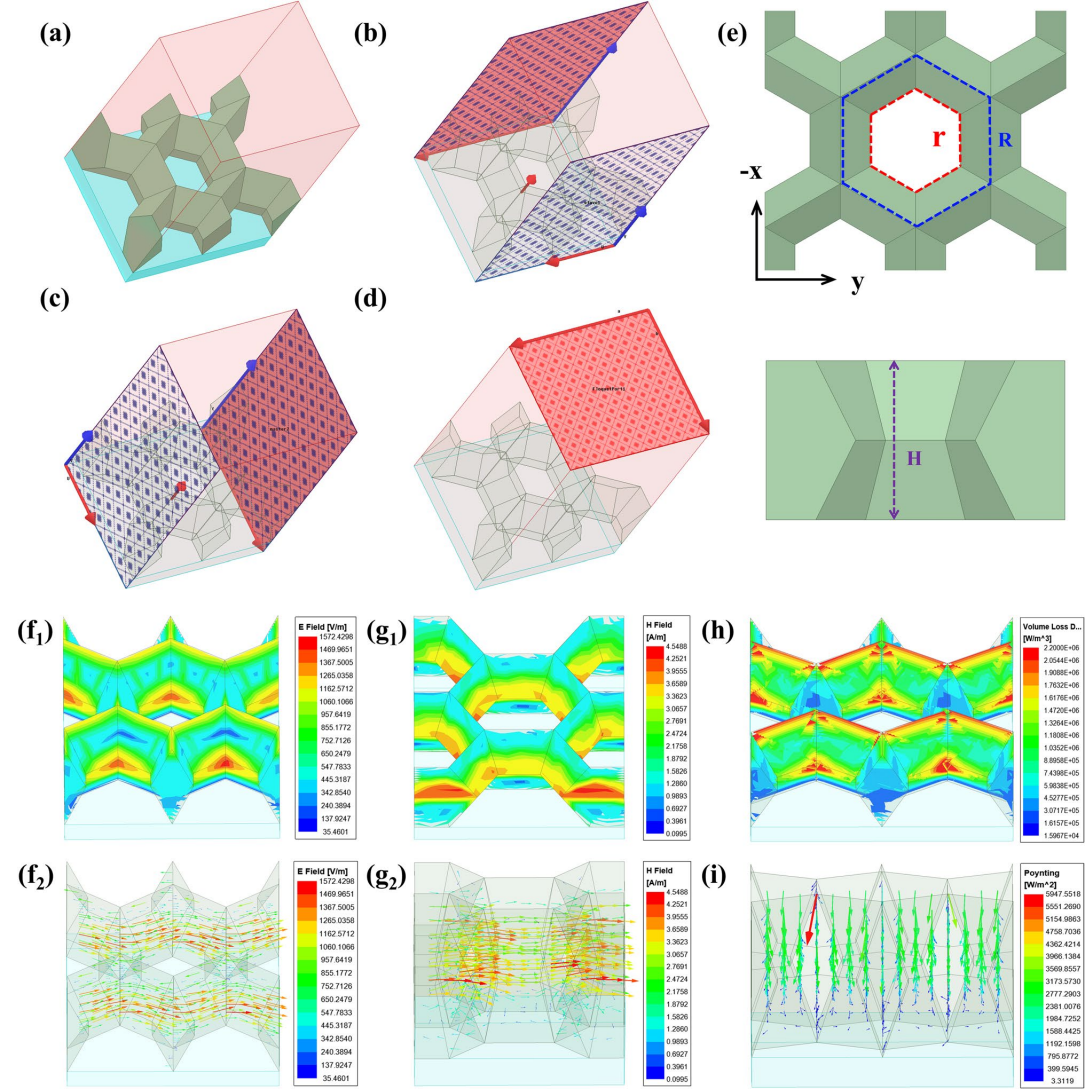
**a** SGHS model; **b**, **c** two sets of master–slave boundaries; **d** sources of excitation for the model; **e** parameters related to the SGHS; **f**_**1**_ Simulated electric field and **f**_**2**_ electric field vector distribution of the SGHS at 10 GHz; **g**_**1**_ Simulated magnetic field and **g**_**2**_ magnetic field vector distribution of the SGHS at 10 GHz; **h** Volume loss density distribution of the SGHS at 10 GHz; **i** Poynting vector distribution of the SGHS at 10 GHz

To further investigate the absorption results of the SGHS, simulations were conducted using HFSS in accordance with the finite element method and transmission line theory. As shown in Fig. 8a, the SGHS model was created with the pink border representing an air box, the bottom blue plate serving as an aluminum plate, and the SGHS in the middle section. Two sets of master–slave boundaries were established (Fig. 8b, c), with the Freud excitation boundary acting as the incident direction and the scanning port serving as the corresponding detection direction (Fig. 8d). During the EM simulation, a periodic structure was assembled through repetition of the honeycomb model. Simultaneously, the EM parameters of MD_3_/PVDF (10 wt%) were imported into the software. EM waves were incident vertically from directly above the honeycomb model. The parameters of the SGHS model are presented in Fig. 8e, including the hexagonal honeycomb side length (R) at the top and bottom of the model, the hexagonal honeycomb side length (r) in the middle of the model, and the thickness (H).

The MA performance of SGHS is determined by its structural features. Therefore, this study focused on investigating the impact of the parameters (H, r, and R) on the MA properties of the SGHS. Thickness (H) has vital importance in honeycomb structures and significantly influences MA properties [57, 58]. As illustrated in Fig. S12a, both the *RL* and EAB of the SGHS increased notably with an increase in thickness. The minimum *RL*_min_ of -59.0 dB was achieved when H = 30 mm, r = 7.5 mm, and R = 9 mm. Figure S12b depicts the impact of r on the MA performance of the SGHS. *RL* strengthened as r decreased within a certain range, but continued reduction in r led to *RL* weakening. This is attributed to the decrease in r causing an increase in the gradient of the honeycomb holes, which, to some extent, promotes impedance matching, enabling greater penetration of EM waves from the upper half [59, 60]. Simultaneously, the significant gradient of the symmetric lower half facilitates better multiple scattering of EM waves, resulting in excellent MA performance. However, excessively small r hinders the entry of EM waves into the SGHS. Meanwhile, EAB increased with decreasing r, continuously expanding the effective absorption of the SGHS at low frequencies. Notably, when r (8.5 mm) and R (9 mm) are nearly equal, the structure resembles the conventional through-hole honeycomb structure, exhibiting poorer MA performance. This indicates a significant advantage of the SGHS over the conventional honeycomb structure. The impact of R on the MA performance of the SGHS aligned with that of r (Fig. S12c). The maximum EAB of 14.6 GHz (3.4 ~ 18.0 GHz) occurred at parameters H = 30 mm, r = 7.5 mm, and R = 13 mm. The gradient of the honeycomb hole is fixed when the difference between R and r is certain. Under the condition of fixed honeycomb hole gradients, the *RL* curves of the structures had the same trend when R and r were varied simultaneously (Fig. S12d). This demonstrates the substantial impact of the honeycomb hole gradient on the MA properties of the SGHS. In summary, the parameters of the SGHS can be chosen based on the particular demands for MA performance.

To further analyze the MA mechanism of the SGHS, Fig. 8 presents the distribution of electric field and vector, magnetic field and vector, volume loss density, and Poynting vector at 10 GHz. In Fig. 8f_1_, in the upper half of the SGHS, the electric field strength increases closer to the honeycomb hole, suggesting that a significant quantity of microwaves penetrates the honeycomb hole. The decreasing gradient of the electric field in the lower half of the SGHS indicates a gradual attenuation of microwaves during transmission. Figure 8f_2_ shows that the local current mainly travels along the wall of the SGHS (y-direction), concentrating primarily above and below the cross section where the honeycomb holes are situated. The geometric properties of the SGHS wall enable a longer path for local current conduction, which is advantageous [57]. The magnetic field distribution was approximated to the electric field distribution, and the results similarly indicated that massive microwaves entered through the honeycomb holes in the upper half of the SGHS and were absorbed in the lower half (Fig. 8g_1_). The direction of the magnetic moments mainly aligned with the honeycomb wall (x-direction, perpendicular to the direction of the local current), and likewise, they predominantly concentrated near the cross section where the honeycomb holes were positioned (Fig. 8g_2_). The results of the volume loss density distribution highlighted that the loss occurs throughout the SGHS (Fig. 8h). The Poynting vector, being an energy flow density vector in the EM field, visualized the distribution of EM energy in the SGHS (Fig. 8i). The results effectively validate the initial design conjecture. The gradient structure of the upper half contributes to the optimization of impedance matching, thereby allowing more microwaves to enter. The symmetric gradient structure of the lower half is better able to confine microwaves, promoting multiple reflection and scattering of the microwaves, extending the transmission path, and depleting EM energy.

## Conclusion

In summary, we systematically synthesized a range of MIL-88C (Fe) precursors with varying AR under diverse preparation conditions, subsequently acquiring the corresponding carbon-coated iron-based derivatives using identical pyrolysis conditions. It is concluded that alterations in the preparation conditions of the MIL-88C (Fe) precursor impact the phases, graphitization degree, and AR of the derivatives, influencing the optimum filling amount, impedance matching, and MA properties. The MD_2_/PVDF, MD_3_/PVDF, and MD_4_/PVDF absorbers demonstrated outstanding MA properties with optimal filler loadings below 20 wt%, some as low as 5 wt%. The MD_2_/PVDF (5 wt%) achieved a maximum EAB of 5.52 GHz (1.90 mm). The MD_3_/PVDF (10 wt%) exhibited a *RL*_min_ value of -67.4 dB at 12.56 GHz (2.13 mm). Additionally, the absorbers prepared in this study displayed minimal SRL values compared to most existing absorbers, signifying stronger RL, thinner thickness, and lower filler content. Through HFSS EM simulation, the integration of the material benefits of MD_3_/PVDF (10 wt%) with the structural advantages of the SGHS resulted in an EAB of 14.6 GHz and a *RL*_min_ of -59.0 dB. It is well suited for the demands of real engineering applications and will prove to be promising candidates in the MA field.

## Supplementary Information

Below is the link to the electronic supplementary material.

Supplementary file1 (DOCX 8091 KB)

## References

[1] Y. Zhang, L. Zhang, L. Tang, R. Du, B. Zhang, S-NiSe/HG nanocomposites with balanced dielectric loss encapsulated in room-temperature self-healing polyurethane for microwave absorption and corrosion protection. ACS Nano. **18**, 8411–8422 (2024). 10.1021/acsnano.3c13057 38436229 10.1021/acsnano.3c13057

[2] Z. Wu, X. Tan, J. Wang, Y. Xing, P. Huang et al., MXene hollow spheres supported by a C-Co exoskeleton grow MWCNTs for efficient microwave absorption. Nano-Micro Lett. **16**, 107 (2024). 10.1007/s40820-024-01326-3 10.1007/s40820-024-01326-3 PMC10837412 38305954

[3] X. Yan, X. Huang, B. Zhong, T. Wu, H. Wang et al., Balancing interface polarization strategy for enhancing electromagnetic wave absorption of carbon materials. Chem. Eng. J. **391**, 123538 (2020). 10.1016/j.cej.2019.123538

[4] L. Liu, H. Deng, X. Tang, Y. Lu, J. Zhou et al., Specific electromagnetic radiation in the wireless signal range increases wakefulness in mice. Proc. Natl. Acad. Sci. U.S.A. **118**, e2105838118 (2021). 10.1073/pnas.2105838118 34330835 10.1073/pnas.2105838118 PMC8346830

[5] K. Zhang, Y. Liu, Y. Liu, Y. Yan, G. Ma et al., Tracking regulatory mechanism of trace Fe on graphene electromagnetic wave absorption. Nano-Micro Lett. **16**, 66 (2024). 10.1007/s40820-023-01280-6 10.1007/s40820-023-01280-6 PMC10767016 38175333

[6] H. Ma, M. Fashandi, Z.B. Rejeb, P. Gong, C.B. Park et al., Efficient electromagnetic wave absorption and thermal infrared stealth in PVTMS@MWCNT nano-aerogel via abundant nano-sized cavities and attenuation interfaces. Nano-Micro Lett. **16**, 20 (2024). 10.1007/s40820-023-01218-y 10.1007/s40820-023-01218-y PMC10656378 37975901

[7] Z. Zhang, Z. Cai, Z. Wang, Y. Peng, L. Xia et al., A review on metal–organic framework-derived porous carbon-based novel microwave absorption materials. Nano-Micro Lett. **13**, 56 (2021). 10.1007/s40820-020-00582-3 10.1007/s40820-020-00582-3 PMC8187524 34138258

[8] L.T. Nguyen, C.J. Goh, T. Bai, R.H. Ong, X.Y. Goh et al., Scalable fabrication of lightweight carbon nanotube aerogel composites for full X-band electromagnetic wave absorption. Carbon **219**, 118811 (2024). 10.1016/j.carbon.2024.118811

[9] Y. Xu, X. Huang, Y. Chen, Y. Liu, X. Long et al., A theoretical strategy of pure carbon materials for lightweight and excellent absorption performance. Carbon **174**, 662–672 (2021). 10.1016/j.carbon.2020.11.044

[10] S.H. Kim, S.Y. Lee, Y. Zhang, S.J. Park, J. Gu, Carbon-based radar absorbing materials toward stealth technologies. Adv. Sci. **10**, 2303104 (2023). 10.1002/advs.202303104 10.1002/advs.202303104 PMC10646258 37735148

[11] Y. Du, Z. Yan, W. You, Q. Men, G. Chen et al., Balancing MXene surface termination and interlayer spacing enables superior microwave absorption. Adv. Funct. Mater. **33**, 2301449 (2023). 10.1002/adfm.202301449

[12] Y. Yan, K. Zhang, G. Qin, T. Zhang, X. Huang, Phase engineering on MoS_2_ to realize dielectric gene engineering for enhancing microwave absorbing performance. Adv. Funct. Mater. (2023). 10.1002/adfm.202316338

[13] D. Pan, G. Yang, H.M. Abo-Dief, V. Murugadoss, N. Naik et al., Vertically aligned silicon carbide nanowires/boron nitride cellulose aerogel networks enhanced thermal conductivity and electromagnetic absorbing of epoxy composites. Nano-Micro Lett. **14**, 118 (2022). 10.1007/s40820-022-00863-z 10.1007/s40820-022-00863-z PMC9056589 35488958

[14] R. Hu, L. Li, X. Xu, D. Pan, J. Dong et al., Dimensional design of Fe_0.9_Co_0.1_ nano-alloys with enhanced low-frequency microwave absorption. Chem. Eng. J. **482**, 148864 (2024). 10.1016/j.cej.2024.148864

[15] M. Yu, S. Li, X. Ren, N. Liu, W. Guo et al., Magnetic bimetallic heterointerface nanomissiles with enhanced microwave absorption for microwave thermal/dynamics therapy of breast cancer. ACS Nano **18**, 3636–3650 (2024). 10.1021/acsnano.3c11433 38227493 10.1021/acsnano.3c11433

[16] R.C. Che, L.M. Peng, X.F. Duan, Q. Chen, X.L. Liang, Microwave absorption enhancement and complex permittivity and permeability of Fe encapsulated within carbon nanotubes. Adv. Mater. **16**, 401–405 (2004). 10.1002/adma.200306460

[17] J.R. Choi, E. Cho, H. Lee, S.B. Lee, W.R. Yu et al., Synthesis of Fe/Co bimetallic metal-organic framework-derived composites and their enhanced electromagnetic wave absorption. Adv Compos Hybrid Mater **7**, 26 (2024). 10.1007/s42114-023-00824-z

[18] S. Surblé, C. Serre, C. Mellot-Draznieks, F. Millange, G. Férey, A new isoreticular class of metal-organic-frameworks with the MIL-88 topology. Chem. Commun. **3**, 284–286 (2006). 10.1039/B512169H 10.1039/b512169h 16391735

[19] C. Serre, C. Mellot-Draznieks, S. Surblé, N. Audebrand, G. Férey et al., Role of solvent-host interactions that lead to very large swelling of hybrid frameworks. Science **315**, 1828–1831 (2007). 10.1126/science.1137975 17395825 10.1126/science.1137975

[20] Y. Dong, X. Zhu, F. Pan, B. Deng, Z. Liu, Mace-like carbon fiber/ZnO nanorod composite derived from Typha orientalis for lightweight and high-efficient electromagnetic wave absorber. Adv. Compos. Hybrid Mater. **4**, 1002–1014 (2021). 10.1007/s42114-021-00277-2

[21] J. Shi, Q. Zhuang, L. Wu, R. Guo, L. Huang et al., Molecular engineering guided dielectric resonance tuning in derived carbon materials. J. Mater. Chem. C **10**, 12257–12265 (2022). 10.1039/D2TC02628G

[22] N. Wu, D. Xu, Z. Wang, F. Wang, J. Liu et al., Achieving superior electromagnetic wave absorbers through the novel metal-organic frameworks derived magnetic porous carbon nanorods. Carbon **145**, 433–444 (2019). 10.1016/j.carbon.2019.01.028

[23] Z. Xiang, Y. Song, J. Xiong, Z. Pan, X. Wang et al., Enhanced electromagnetic wave absorption of nanoporous Fe_3_O_4_ @ carbon composites derived from metal-organic frameworks. Carbon **142**, 20–31 (2019). 10.1016/j.carbon.2018.10.014

[24] Z.X. Liu, H.B. Yang, Z.M. Han, W.B. Sun, X.X. Ge et al., A bioinspired gradient design strategy for cellulose-based electromagnetic wave absorbing structural materials. Nano Lett. **24**, 881–889 (2024). 10.1021/acs.nanolett.3c03989 38198246 10.1021/acs.nanolett.3c03989

[25] Z. Zhang, H. Lei, S. Duan, Z. Zhao, M. Chen et al., Bioinspired double-broadband switchable microwave absorbing grid structures with inflatable kresling origami actuators. Adv. Sci. **11**, 2306119 (2024). 10.1002/advs.202306119 10.1002/advs.202306119 PMC10811514 38036422

[26] D.D. Lim, S. Lee, J.H. Lee, W. Choi, G.X. Gu, Mechanical metamaterials as broadband electromagnetic wave absorbers: investigating relationships between geometrical parameters and electromagnetic response. Mater. Horiz. (2024). 10.1039/D3MH01959D 10.1039/d3mh01959d 38477233

[27] H. Yan, S. Xuan, X. Fan, Y. Shan, X. Xu et al., A repair efficiency evaluation framework for the honeycomb microwave absorbing structure. Compos. Sci. Technol. **248**, 110471 (2024). 10.1016/j.compscitech.2024.110471

[28] C. Wang, S. Ma, D. Li, J. Zhao, H. Zhou et al., Direct ink writing of thermoresistant, lightweight composite polyimide honeycombs with tunable X-band electromagnetic wave absorption properties. Addit. Manuf. **70**, 103554 (2023). 10.1016/j.addma.2023.103554

[29] G. Lee, S. Lee, S. Oh, D. Kim, M. Oh, Tip-to-middle anisotropic MOF-on-MOF growth with a structural adjustment. J. Am. Chem. Soc. **142**, 3042–3049 (2020). 10.1021/jacs.9b12193 31968935 10.1021/jacs.9b12193

[30] C. Serre, F. Millange, S. Surblé, G. Férey, A route to the synthesis of trivalent transition-metal porous carboxylates with trimeric secondary building units. Angew. Chem. Int. Ed. **116**, 6445–6449 (2004). 10.1002/ange.200454250 10.1002/anie.200454250 15372643

[31] Y. Ding, Z.A. Qiao, Carbon surface chemistry: new insight into the old story. Adv. Mater. **34**, 2206025 (2022). 10.1002/adma.202206025 10.1002/adma.202206025 36127265

[32] Y. Zhang, C.M. Liang, M. Lu, H. Yu, G.S. Wang, Skillful introduction of urea during the synthesis of MOF-derived FeCoNi–CH/p-rGO with a spindle-shaped substrate for hybrid supercapacitors. ACS Omega **7**, 33019–33030 (2022). 10.1021/acsomega.2c02712 36157736 10.1021/acsomega.2c02712 PMC9494635

[33] Y. Tang, J. Ding, W. Zhou, S. Cao, F. Yang et al., Design of uniform hollow carbon nanoarchitectures: different capacitive deionization between the hollow shell thickness and cavity size. Adv. Sci. **10**, 2206960 (2023). 10.1002/advs.202206960 10.1002/advs.202206960 PMC10037972 36658723

[34] P.Y. Zhao, H.Y. Wang, B. Cai, X.B. Sun, Z.L. Hou et al., Electrospinning fabrication and ultra-wideband electromagnetic wave absorption properties of CeO_2_/N-doped carbon nanofibers. Nano Res. **15**, 7788–7796 (2022). 10.1007/s12274-022-4675-x

[35] B. Sayahpour, H. Hirsh, S. Bai, N. Schorr, T. Lambert et al., Revisiting discharge mechanism of CF_x_ as a high energy density cathode material for lithium primary battery. Adv. Energy Mater. **12**, 2103196 (2022). 10.1002/aenm.202103196

[36] H. Xu, X. Yin, X. Fan, Z. Tang, Z. Hou et al., Constructing a tunable heterogeneous interface in bimetallic metal-organic frameworks derived porous carbon for excellent microwave absorption performance. Carbon **148**, 421–429 (2019). 10.1016/j.carbon.2019.03.091

[37] Q. Chang, H. Liang, B. Shi, H. Wu, Microstructure induced dielectric loss in lightweight Fe_3_O_4_ foam for electromagnetic wave absorption. iScience **25**, 103925 (2022). 10.1016/j.isci.2022.103925 35252818 10.1016/j.isci.2022.103925 PMC8889371

[38] N. Tsumori, L. Chen, Q. Wang, M. Kitta, Q. Xu et al., Quasi-MOF: exposing inorganic nodes to guest metal nanoparticles for drastically enhanced catalytic activity. Chem **4**, 845–856 (2018). 10.1016/j.chempr.2018.03.009

[39] H.Y. Wang, X.B. Sun, Y. Xin, S.H. Yang, P.F. Hu et al., Ultrathin self-assembly MXene/Co-based bimetallic oxide heterostructures as superior and modulated microwave absorber. J. Mater. Sci. Technol. **134**, 132–141 (2023). 10.1016/j.jmst.2022.05.061

[40] X.J. Zhang, G.S. Wang, Y.Z. Wei, L. Guo, M.S. Cao, Polymer-composite with high dielectric constant and enhanced absorption properties based on graphene-CuS nanocomposites and polyvinylidene fluoride. J. Mater. Chem. A **1**, 12115–12122 (2013). 10.1039/C3TA12451G

[41] H.Y. Wang, X.B. Sun, S.H. Yang, P.Y. Zhao, X.J. Zhang et al., 3D ultralight hollow NiCo compound@MXene composites for tunable and high-efficient microwave absorption. Nano-Micro Lett. **13**, 206 (2021). 10.1007/s40820-021-00727-y 10.1007/s40820-021-00727-y PMC8505608 34633551

[42] S. Gao, G.S. Wang, L. Guo, S.H. Yu, Tunable and ultraefficient microwave absorption properties of trace N-doped two-dimensional carbon-based nanocomposites loaded with multi-rare earth oxides. Small **16**, 1906668 (2020). 10.1002/smll.201906668 10.1002/smll.201906668 32297713

[43] Z.L. Hou, X. Gao, J. Zhang, G. Wang, A perspective on impedance matching and resonance absorption mechanism for electromagnetic wave absorbing. Carbon **222**, 118935 (2024). 10.1016/j.carbon.2024.118935

[44] M. Tang, J.Y. Zhang, S. Bi, Z.L. Hou, X.H. Shao et al., Ultrathin topological insulator absorber: unique dielectric behavior of Bi_2_Te_3_ nanosheets based on conducting surface states. ACS Appl. Mater. Interfaces **11**, 33285–33291 (2019). 10.1021/acsami.9b13775 10.1021/acsami.9b13775 31429548

[45] X. Zhou, J. Wen, Z. Wang, X. Ma, H. Wu, Broadband high-performance microwave absorption of the single-layer Ti_3_C_2_T_x_ MXene. J. Mater. Sci. Technol. **115**, 148–155 (2022). 10.1016/j.jmst.2021.11.029

[46] X. Zhou, B. Wang, Z. Jia, X. Zhang, X. Liu et al., Dielectric behavior of Fe_3_N@C composites with green synthesis and their remarkable electromagnetic wave absorption performance. J. Colloid Interface Sci. **582**, 515–525 (2021). 10.1016/j.jcis.2020.08.087 32911400 10.1016/j.jcis.2020.08.087

[47] L. Deng, M. Han, Microwave absorbing performances of multiwalled carbon nanotube composites with negative permeability. Appl. Phys. Lett. **91**, 023119 (2007). 10.1063/1.2755875

[48] H.Y. Wang, X.B. Sun, G.S. Wang et al., A MXene-modulated 3D crosslinking network of hierarchical flower-like MOF derivatives towards ultra-efficient microwave absorption properties. J. Mater. Chem. A **9**, 24571–24581 (2021). 10.1039/D1TA06505J

[49] G. Wu, Y. Cheng, Z. Yang, Z. Jia, H. Wu et al., Design of carbon sphere/magnetic quantum dots with tunable phase compositions and boost dielectric loss behavior. Chem. Eng. J. **333**, 519–528 (2018). 10.1016/j.cej.2017.09.174

[50] Z. Tang, L. Xu, C. Xie, L. Guo, L. Zhang et al., Synthesis of CuCo_2_S_4_@expanded graphite with crystal/amorphous heterointerface and defects for electromagnetic wave absorption. Nat. Commun. **14**, 5951 (2023). 10.1038/s41467-023-41697-6 37741860 10.1038/s41467-023-41697-6 PMC10517935

[51] M. Qin, L. Zhang, H. Wu, Dielectric loss mechanism in electromagnetic wave absorbing materials. Adv. Sci. **9**, 2105553 (2022). 10.1002/advs.202105553 10.1002/advs.202105553 PMC8981909 35128836

[52] Y. Liu, X. Zhou, Z. Jia, H. Wu, G. Wu, Oxygen vacancy-induced dielectric polarization prevails in the electromagnetic wave-absorbing mechanism for Mn-based MOFs-derived composites. Adv. Funct. Mater. **32**, 2204499 (2022). 10.1002/adfm.202204499

[53] Y. Liu, X. Wei, X. He, J. Yao, R. Tan et al., Multifunctional shape memory composites for Joule heating, self-healing, and highly efficient microwave absorption. Adv. Funct. Mater. **33**, 2211352 (2022). 10.1002/adfm.202211352

[54] X.J. Zhang, J.Q. Zhu, P.G. Yin, A.P. Guo, A.P. Huang et al., Tunable high-performance microwave absorption of Co_1–x_S hollow spheres constructed by nanosheets within ultralow filler loading. Adv. Funct. Mater. **28**, 1800761 (2018). 10.1002/adfm.201800761

[55] M. Green, Z. Liu, P. Xiang, X. Tan, F. Huang et al., Ferric metal-organic framework for microwave absorption. Mater. Today Chem. **9**, 140–148 (2018). 10.1016/j.mtchem.2018.06.003

[56] N. Yang, Z.X. Luo, G.R. Zhu, S.C. Chen, X.L. Wang et al., Ultralight three-dimensional hierarchical cobalt nanocrystals/N-doped CNTs/carbon sponge composites with hollow skeleton toward superior microwave absorption. ACS Appl. Mater. Interfaces **11**, 35987–35998 (2019). 10.1021/acsami.9b11101 31496213 10.1021/acsami.9b11101

[57] H. Pang, Y. Duan, X. Dai, L. Huang, X. Yang et al., The electromagnetic response of composition-regulated honeycomb structural materials used for broadband microwave absorption. J. Mater. Sci. Technol. **88**, 203–214 (2021). 10.1016/j.jmst.2021.01.072

[58] N. Zhang, M. Han, G. Wang, Y. Zhao, W. Gu et al., Achieving broad absorption bandwidth of the Co/carbon absorbers through the high-frequency structure simulator electromagnetic simulation. J. Alloy. Compd. **883**, 160918 (2021). 10.1016/j.jallcom.2021.160918

[59] H. Yan, B. Fu, S. Xuan, T. Qin, X. Yao, Electromagnetic response of grading honeycomb composites for broadband microwave absorption. Compos. Struct. **321**, 117280 (2023). 10.1016/j.compstruct.2023.117280

[60] F. Luo, D. Liu, T. Cao, H. Cheng, J. Kuang et al., Study on broadband microwave absorbing performance of gradient porous structure. Adv. Compos. Hybrid Mater. **4**, 591–601 (2021). 10.1007/s42114-021-00275-4

