# Supplementary material for: Designing Symmetric Gradient Honeycomb Structures with Carbon-Coated Iron-Based Composites for High-Efficiency Microwave Absorption
Source: Nanomicro Lett. 2024 Jul 2;16:234. doi: 10.1007/s40820-024-01435-z (PMC11219676; doi:10.1007/s40820-024-01435-z)
Supplement: Supplementary file 1 — Supplementary file1 (DOCX 8091 KB) [file 40820_2024_1435_MOESM1_ESM.docx]

Supporting Information for

**Designing Symmetric Gradient Honeycomb Structures with Carbon-Coated Iron-Based Composites for High-Efficiency Microwave Absorption**

**Supplementary Figures and Tables**


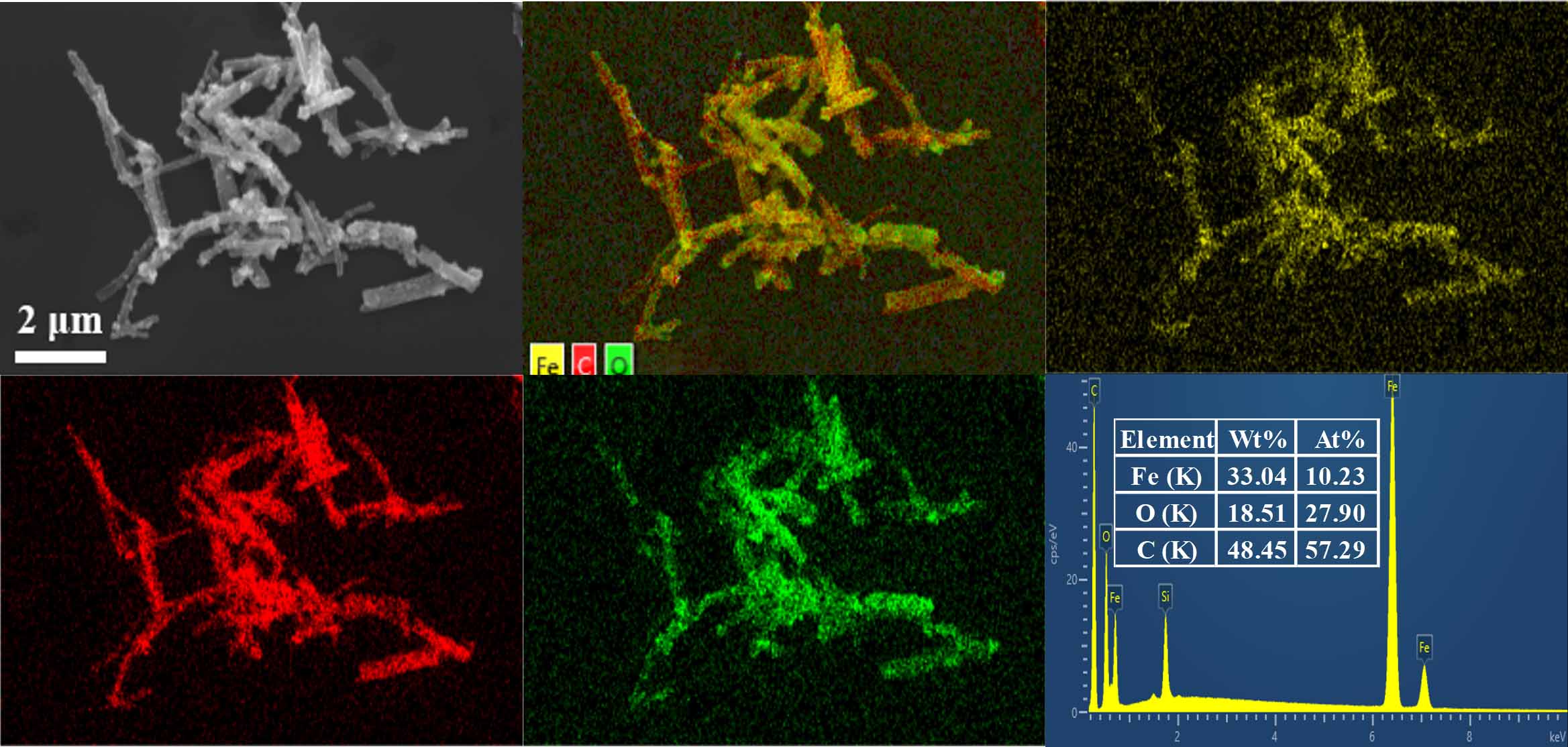


**Fig. S1** EDS element mapping images and spectra of MD_3_


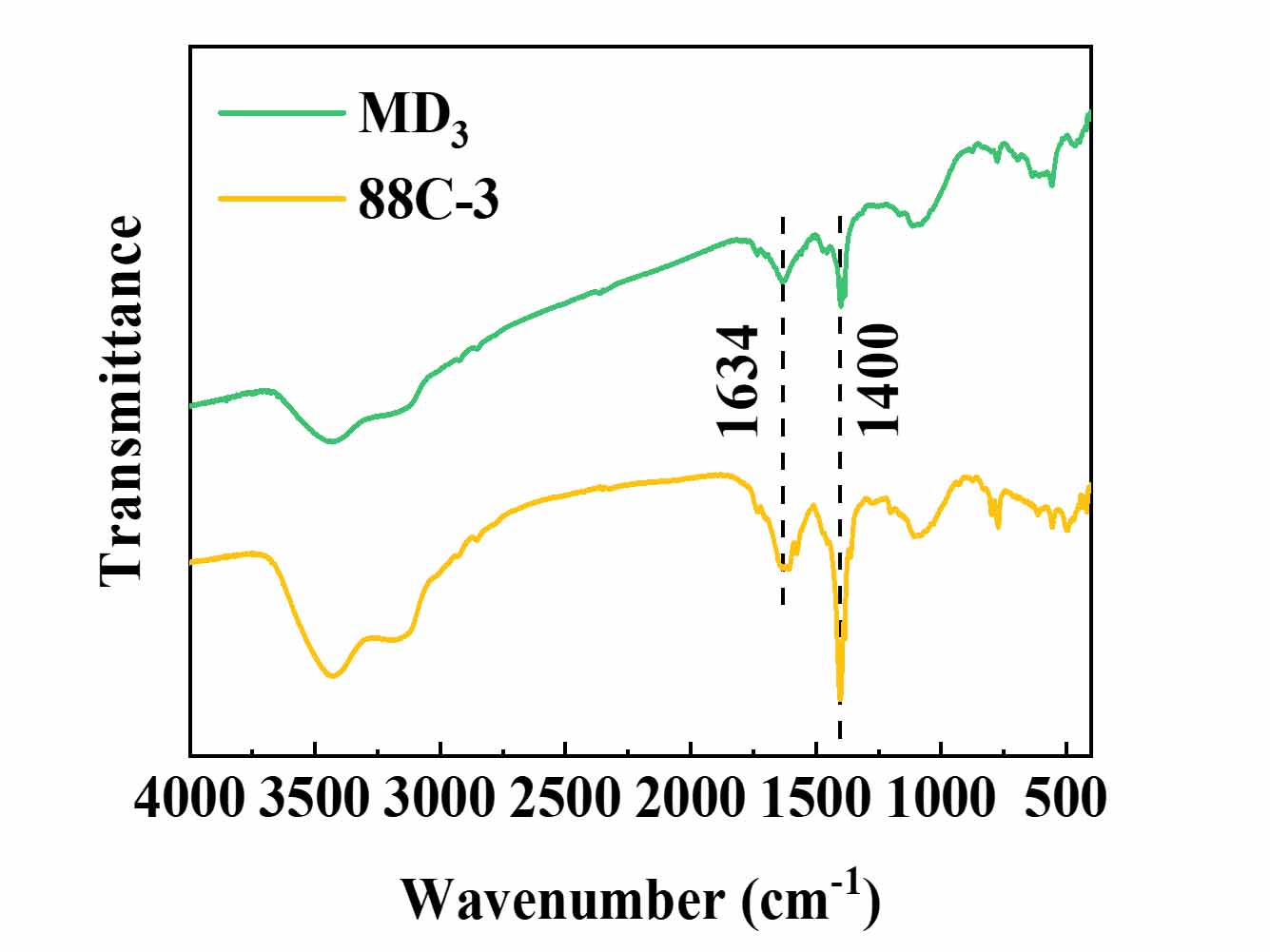


**Fig. S2** FT-IR spectra of 88C-3 and MD_3_

_
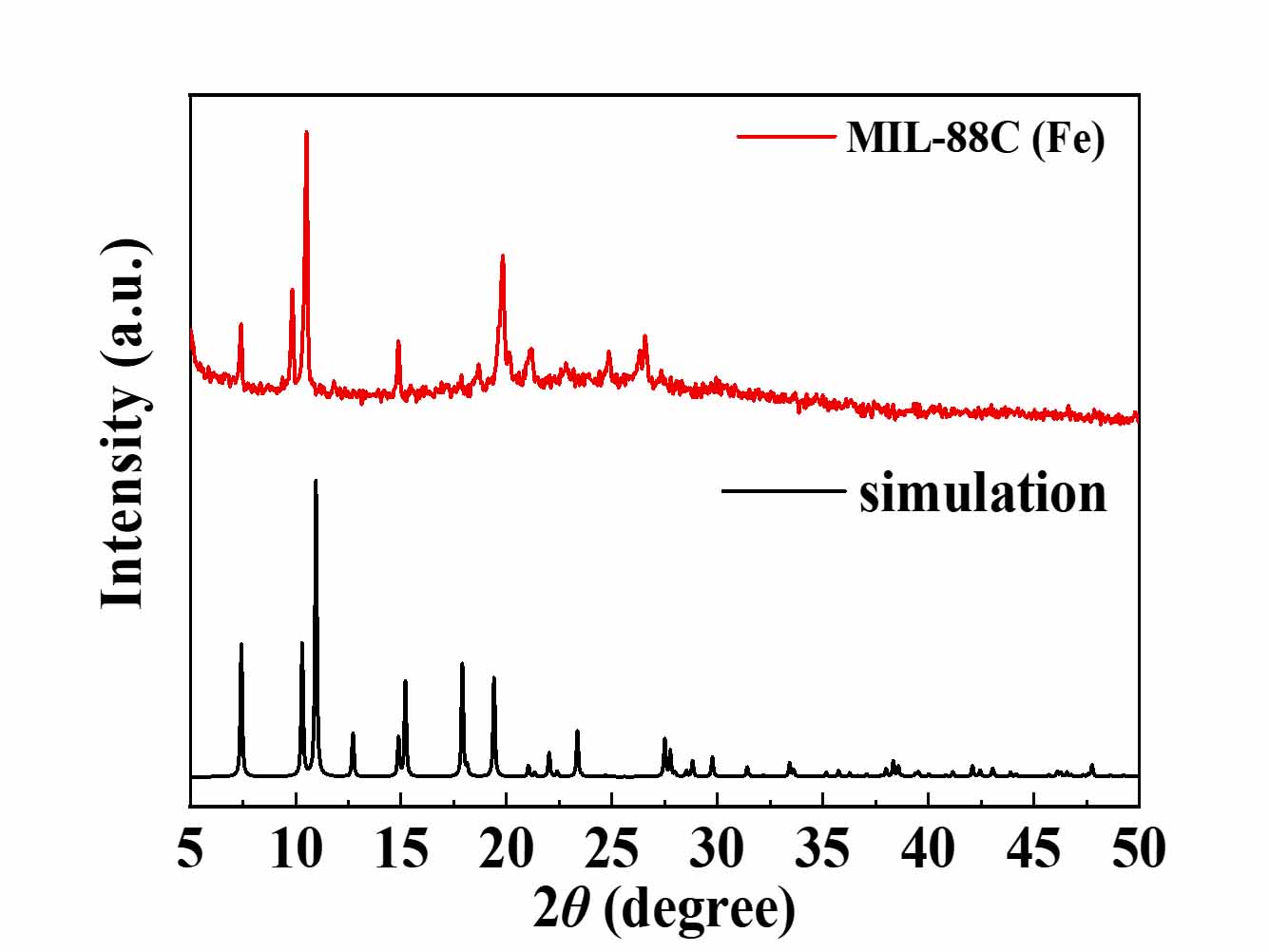
_

**Fig. S3** XRD pattern of MIL-88C (Fe) taking 88C-2 as an example


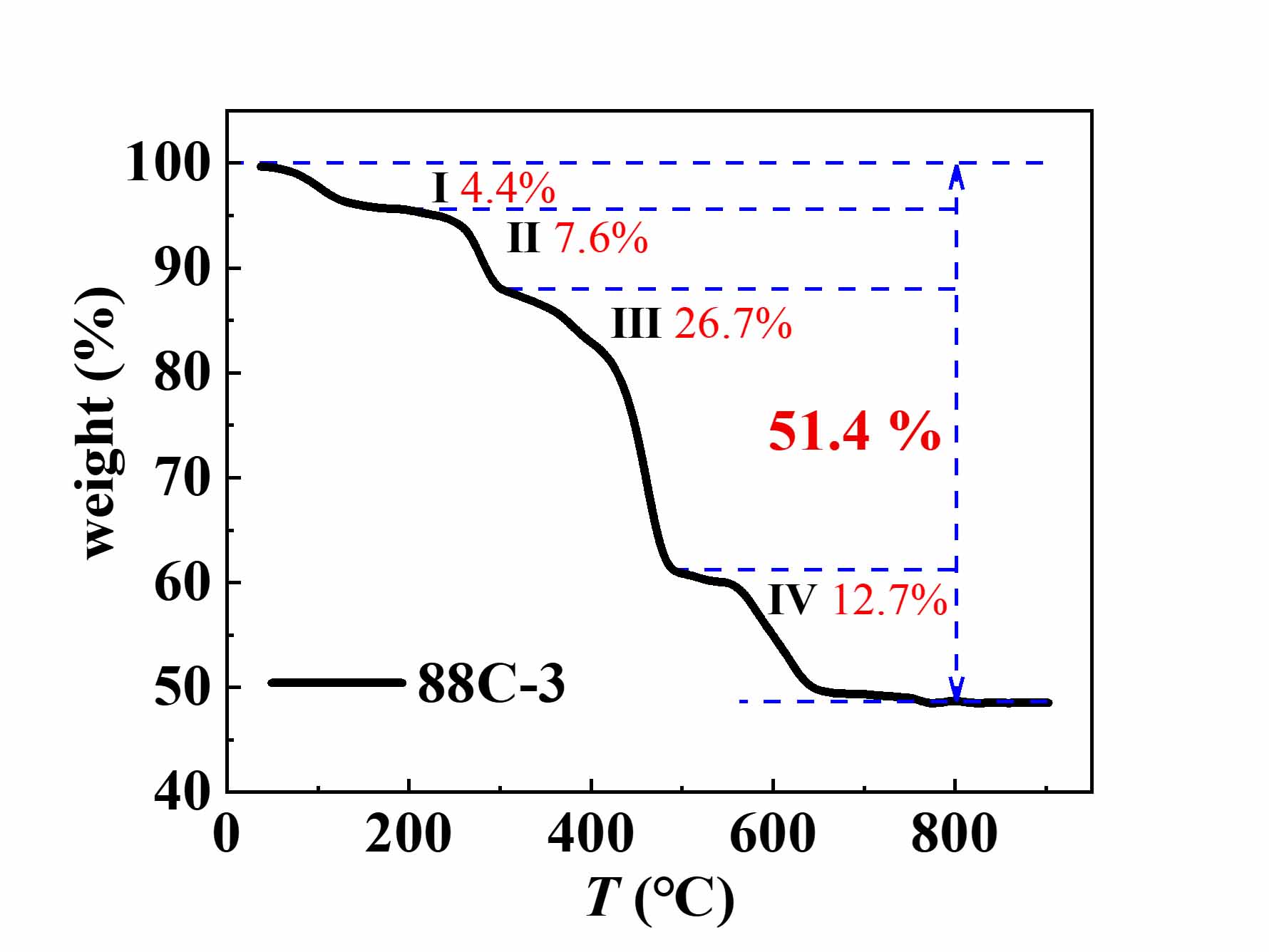


**Fig. S4** TG curve of 88C-3 under N_2_ atmosphere


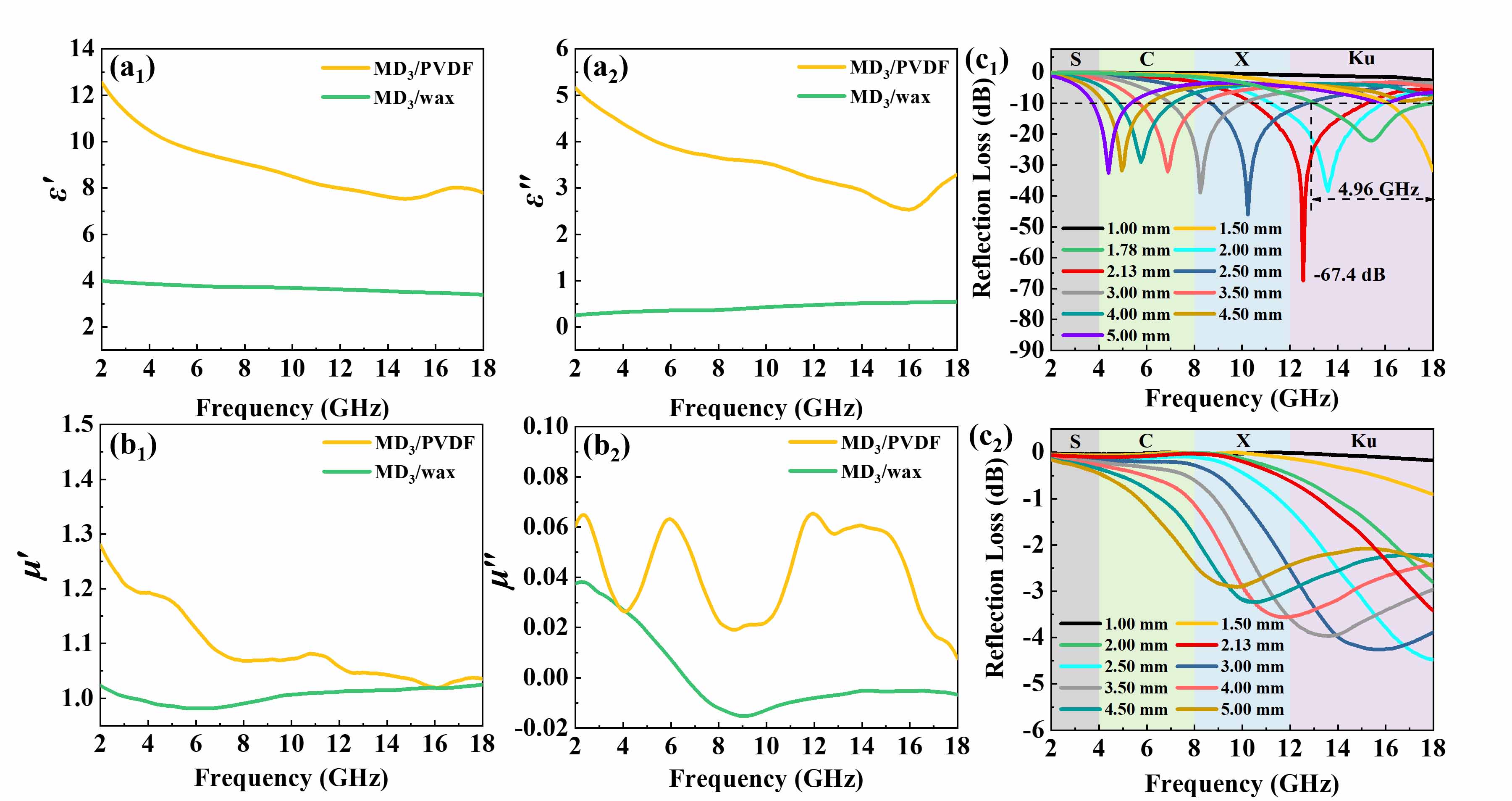


**Fig. S5** Dielectric characterization: **a_1_** real part *ε'*, **a_2_** imaginary part *ε''*; Magnetic characterization: **b_1_** real part *μ'*, **b_2_** imaginary part *μ''* of samples; 2D RL curves of **c_1_** MD_3_/PVDF and **c_2_** MD_3_/wax with filler loading of 10 wt%


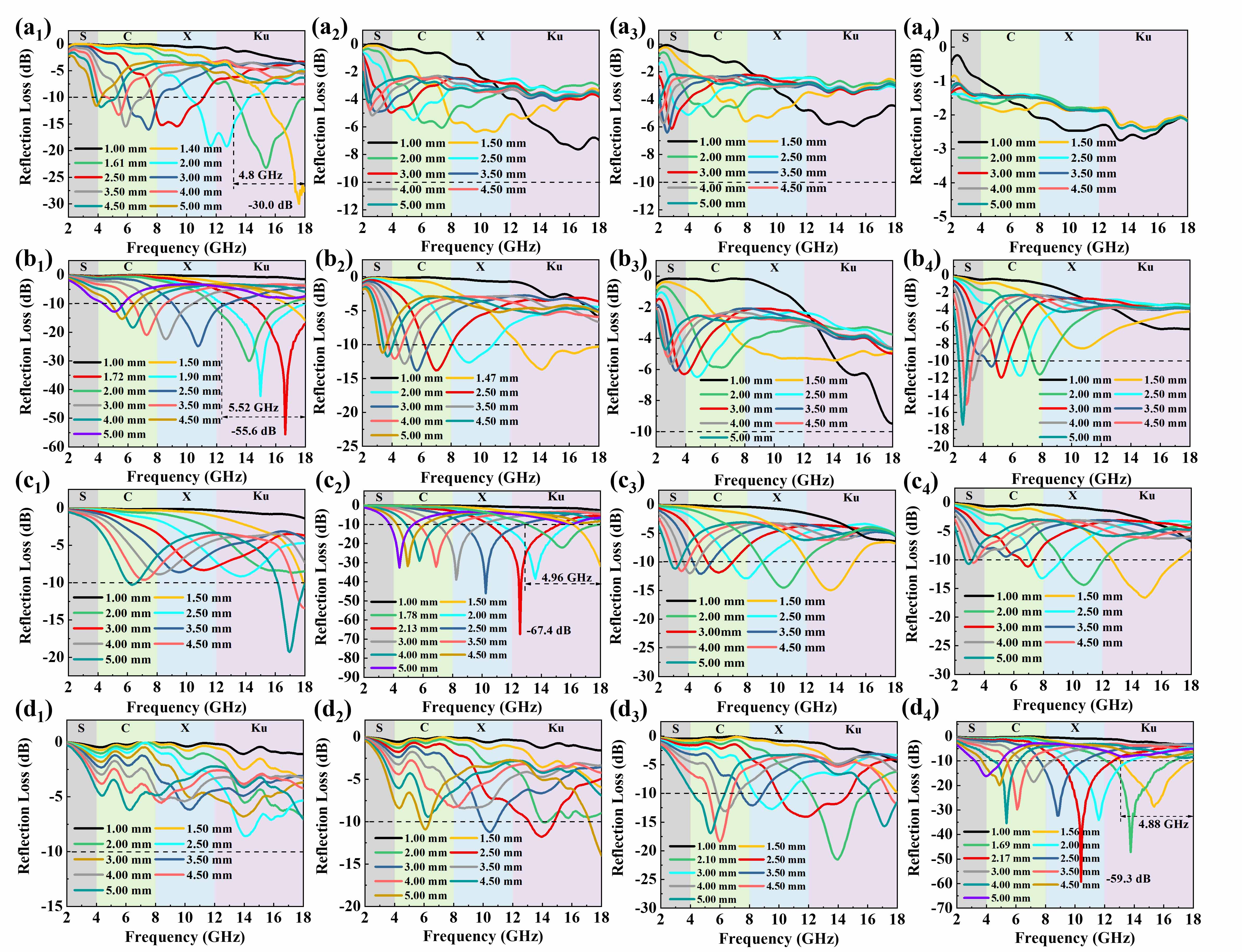


**Fig. S6** 2D RL curves of MD_1_/PVDF at filler loading of **a_1_** 5 wt%, **a_2_** 10 wt%, **a_3_** 15 wt%, and **a_4_** 20 wt%; MD_2_/PVDF at filler loading of **b_1_** 5 wt%, **b_2_** 10 wt%, **b_3_** 15 wt%, and **b_3_** 20 wt%; MD_3_/PVDF at filler loading of **c_1_** 5 wt%, **c_2_** 10 wt%, **c_3_** 15 wt%, and **c_4_** 20 wt%; MD_4_/PVDF at filler loading of **d_1_** 5 wt%, **d_2_** 10 wt%, **d_3_** 15 wt%, and **d_4_** 20 wt%


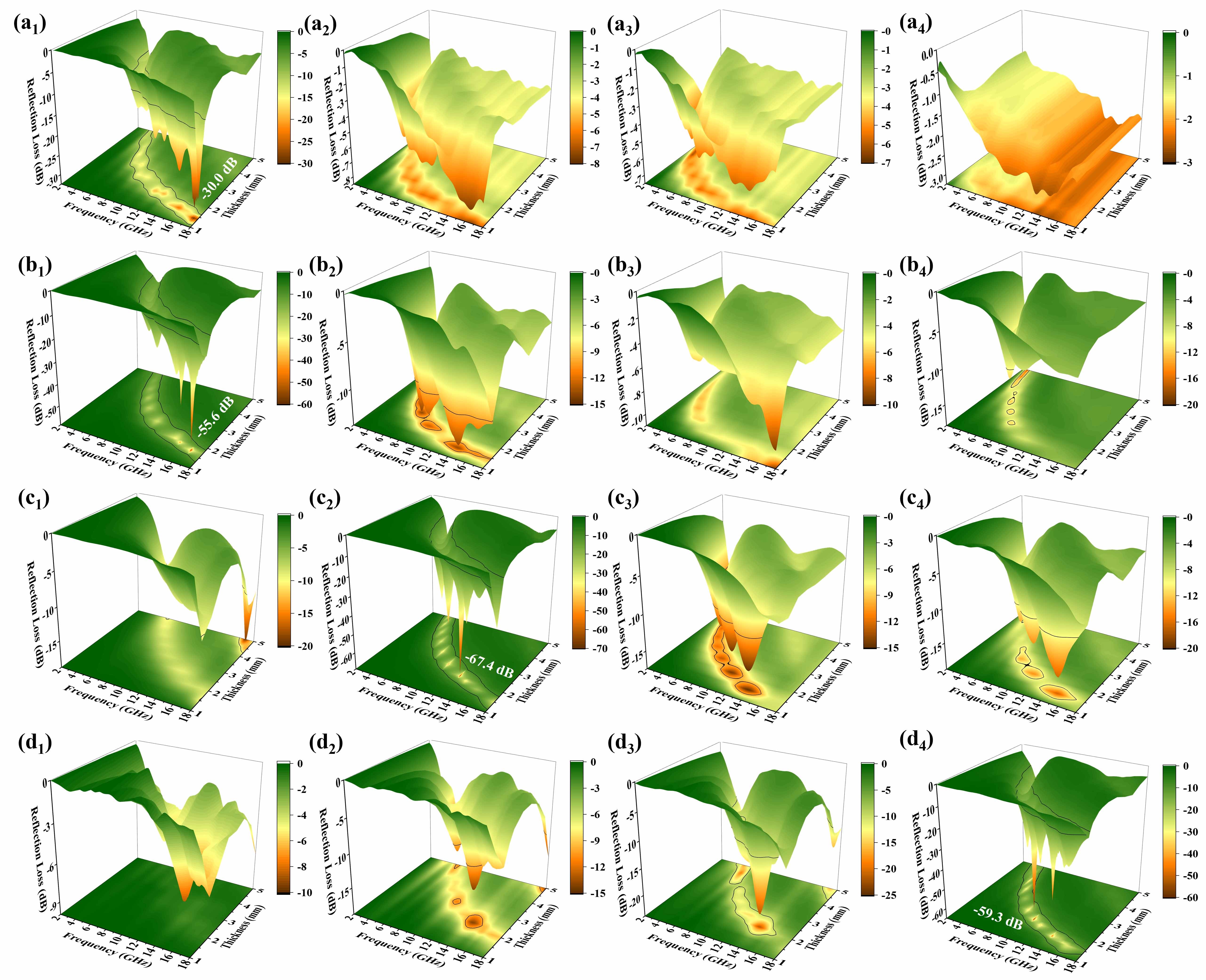


**Fig. S7** 3D RL curves of MD_1_/PVDF at filler loading of **a_1_** 5 wt%, **a_2_** 10 wt%, **a_3_** 15 wt%, and **a_4_** 20 wt%; MD_2_/PVDF at filler loading of **b_1_** 5 wt%, **b_2_** 10 wt%, **b_3_** 15 wt%, and **b_3_** 20 wt%; MD_3_/PVDF at filler loading of **c_1_** 5 wt%, **c_2_** 10 wt%, **c_3_** 15 wt%, and **c_4_** 20 wt%; MD_4_/PVDF at filler loading of **d_1_** 5 wt%, **d_2_** 10 wt%, **d_3_** 15 wt%, and **d_4_** 20 wt%


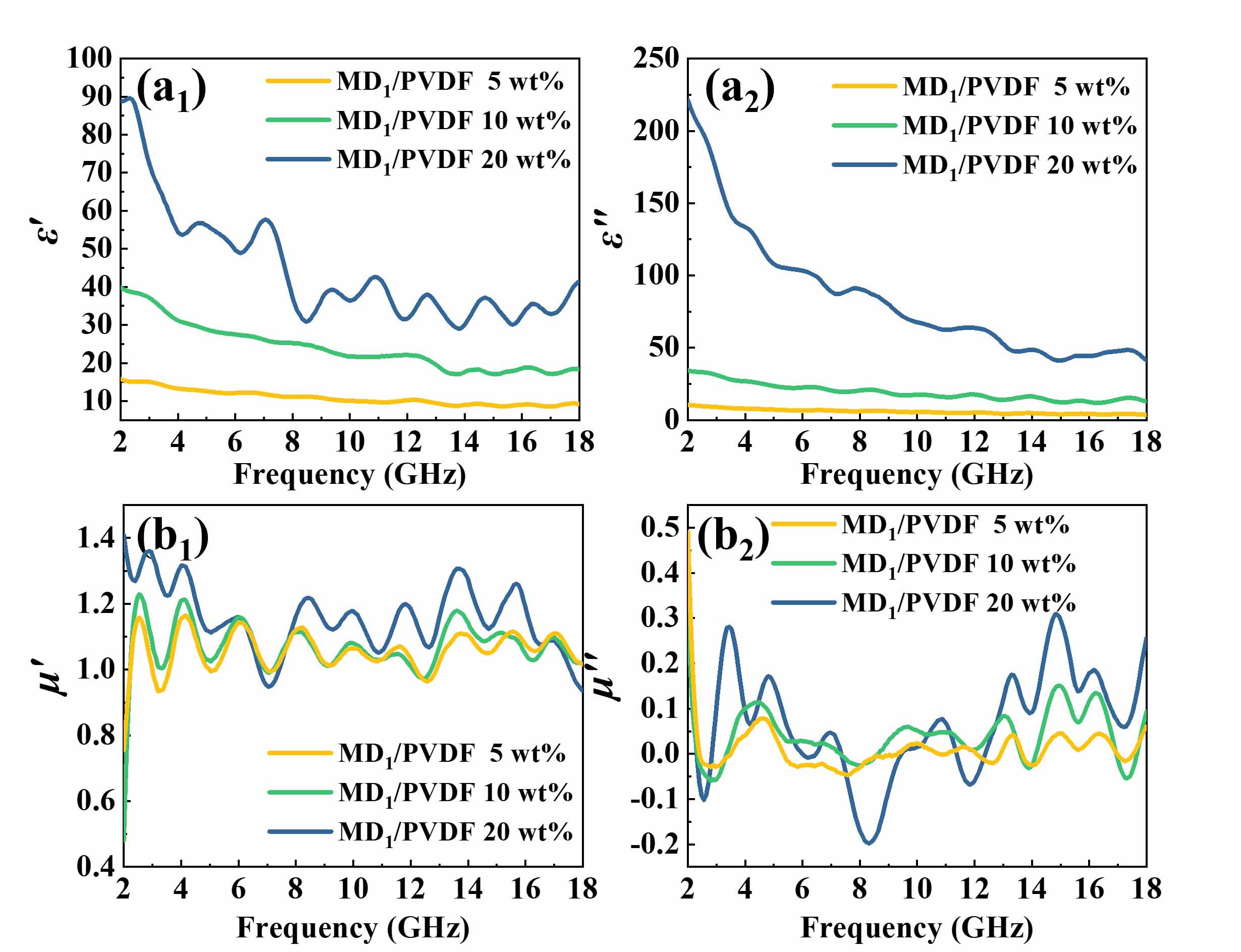


**Fig. S8** Dielectric characterization: **a_1_** real part *ε'*, **a_2_** imaginary part *ε''*; Magnetic characterization: **b_1_** real part *μ'*, **b_2_** imaginary part *μ''* of MD_1_/PVDF with various filler loading of 5 wt%, 10 wt%, and 20 wt%


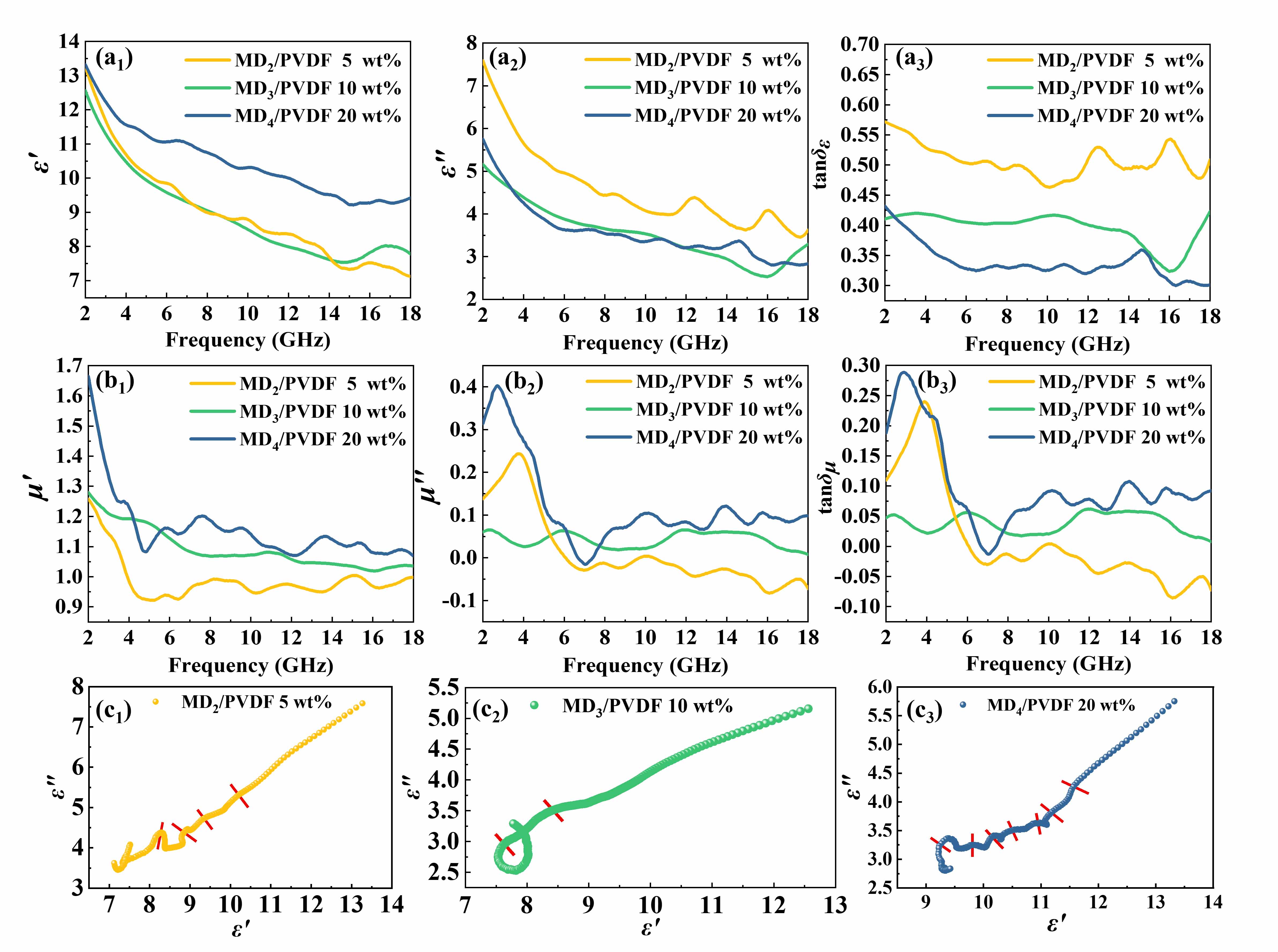


**Fig. S9** Dielectric characterization: **a_1_** real part *ε'*, **a_2_** imaginary part *ε''*, and **a_3_** dielectric loss tangent; Magnetic characterization: **b_1_** real part *μ'*, **b_2_** imaginary part *μ''*, and **b_3_** magnetic loss tangent; **c_1_~c_3_** relationship between real and imaginary dielectric part of the MD_2_/PVDF (5 wt%), MD_3_/PVDF (10 wt%), and MD_4_/PVDF (20 wt%)


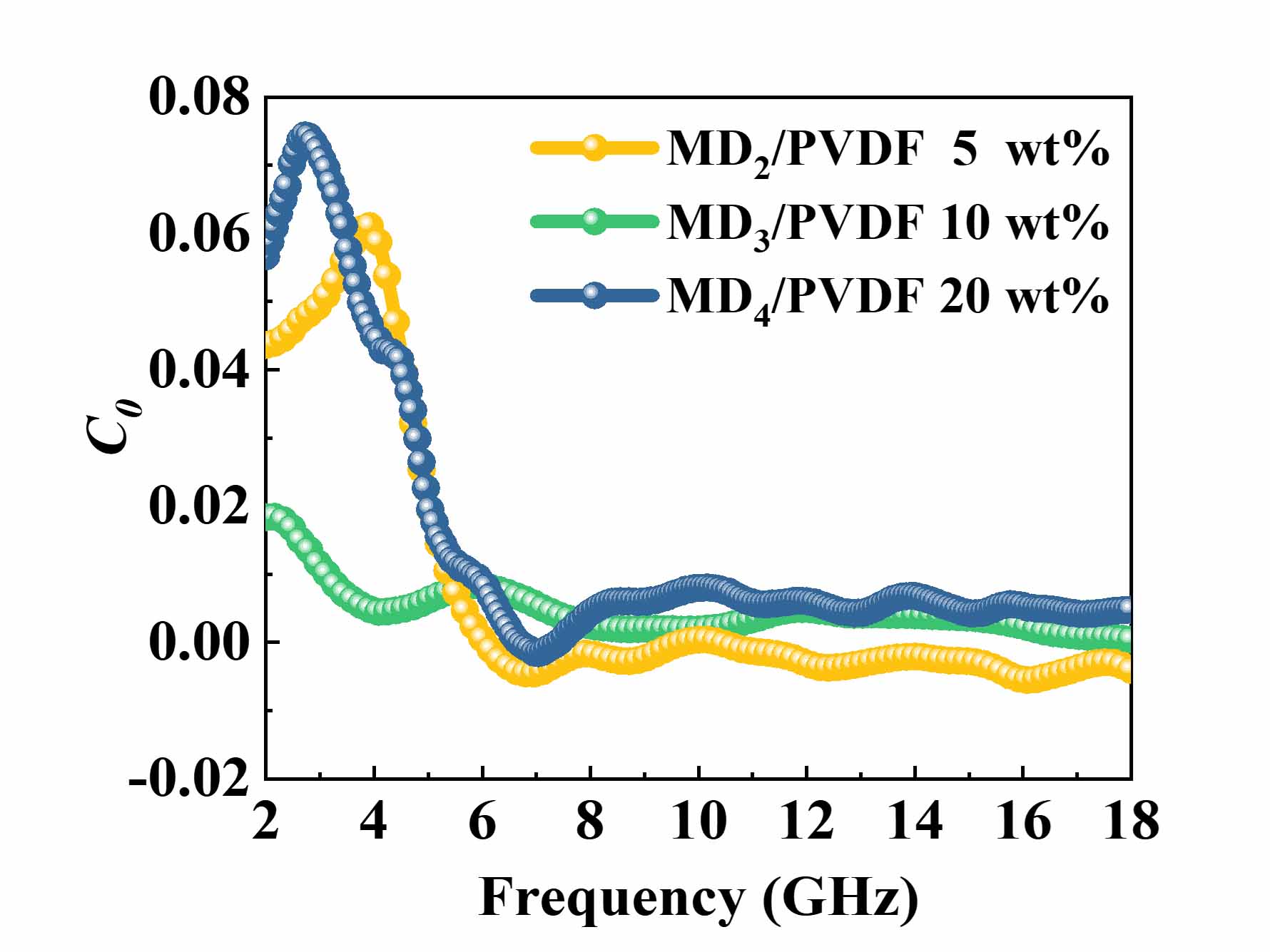


**Fig. S10** *C_0_* values of the MD_2_/PVDF (5 wt%), MD_3_/PVDF (10 wt%), and MD_4_/PVDF (20 wt%), respectively


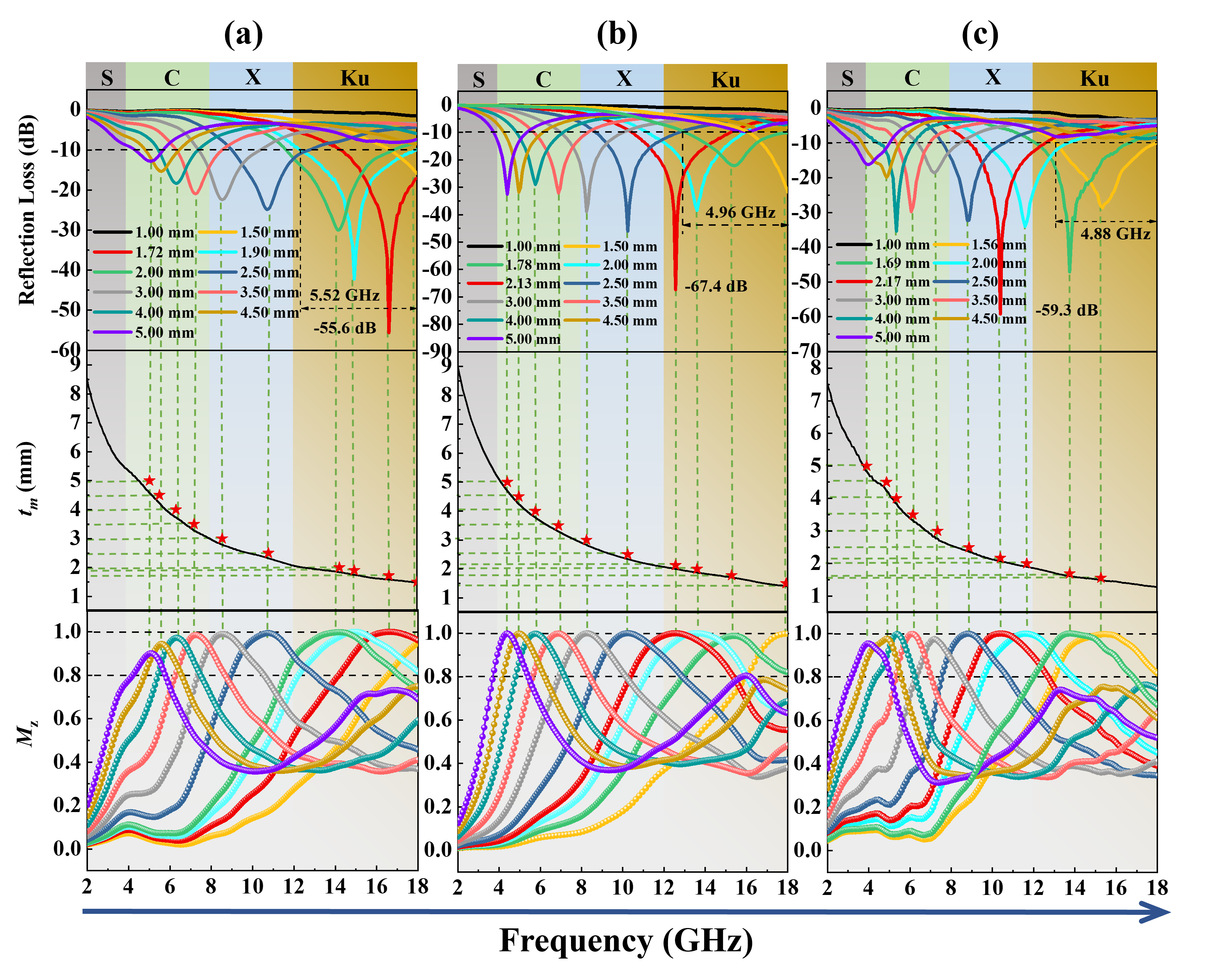


**Fig. S11** 2D RL, dependence of *t_m_* on *f_m_* with the λ/4 model and *M*_z_ with various thicknesses for **a** MD_2_/PVDF (5 wt%), **b** MD_3_/PVDF (10 wt%), and **c** MD_4_/PVDF (20 wt%)


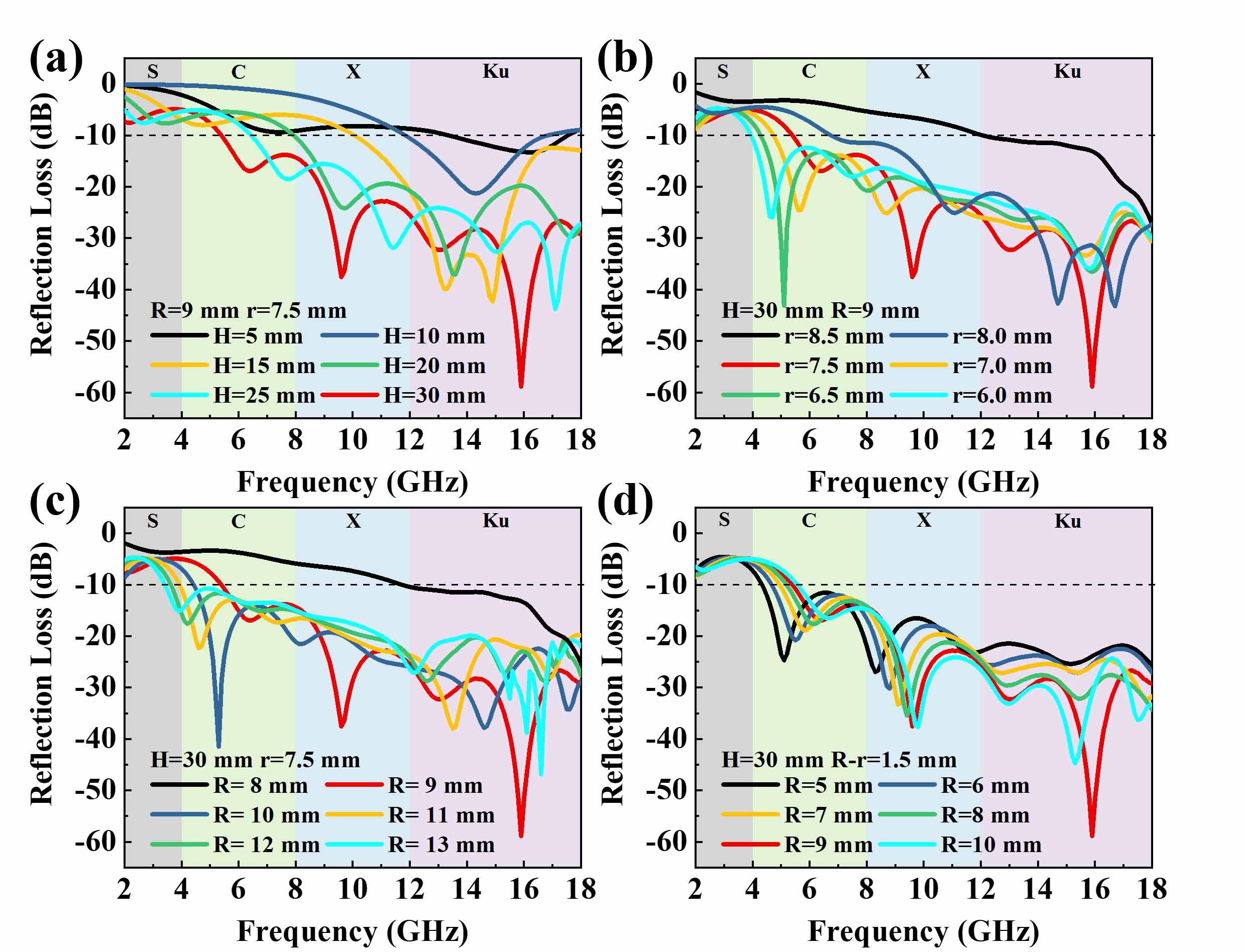


**Fig. S12** The MA performance of the modified honeycomb structure at **a** H of 5~30 mm, **b** r of 6.0~8.5 mm, **c** R of 8~13 mm, and **d** R/r of 5/3.5~10/8.5 mm

**Table S1** MA properties of previously reported absorbers and this work

| Sample | Matrix | Filler loading (wt%) | Optimal RL /Matching thickness (dB/mm) | Maximum EAB /Matching thickness  (GHz/mm) | Reference | |
| --- | --- | --- | --- | --- | --- | --- |
|  |  |  |  |  |  |  |
| Fe_3_O_4_@NPC  derived from  MIL-88A (Fe) | epoxy  resin | 40 | -59.2 (1.58) | 4.64 (3.07) | [S1] | |
| Fe_3_O_4_@NPC  derived from  MIL-88B (Fe) | wax | 40 | -65.5 (3.0) | 4.5 (3.0) | [S2] | |
| CN-Fe_3_C | PVDF | 10 | -46.8 (2.0) | 5.01 (2.0) | [S3] | |
| Fe_3_S_4_@C | wax | 20 | -58.9 (3.1) | 5.4 (2) | [S4] | |
| PC/Fe | wax | 40 | -48.4 (2.245) | 4.2 (1.5) | [S5] | |
| CoFe@PCS | wax | 15 | -57.7 (2.0) | 4.2 (2.0) | [S6] | |
| NiSe_2_-FeSe@NC | wax | 45 | -52.8 (2.1) | 4.5 (1.7) | [S7] | |
| NiFe_2_O_4_/FeNi_3_@EG | wax | 5 | -39.1 (3.0) | 4.0 (1.5) | [S8] |  |
| Fe/Fe_3_O_4_@C  (MD_2_) | PVDF | 5 | -55.6 (1.72) | 5.52 (1.9) | This work | |
| Fe_3_O_4_@C  (MD_3_) | PVDF | 10 | -67.4 (2.13) | 4.96 (1.78) | This work | |

**Supplementary References**

S1 N. Wu, D. Xu, Z. Wang, F. Wang, J. Liu et al., Achieving superior electromagnetic wave absorbers through the novel metal-organic frameworks derived magnetic porous carbon nanorods. Carbon **145**, 433–444 (2019). https://doi.org/10.1016/j.carbon.2019.01.028

S2 Z. Xiang, Y. Song, J. Xiong, Z. Pan, X. Wang et al., Enhanced electromagnetic wave absorption of nanoporous Fe_3_O_4_@carbon composites derived from metal-organic frameworks. Carbon **142**, 20–31 (2019). <https://doi.org/10.1016/j.carbon.2018.10.014>

S3 S. Gao, S.H. Yang, H.Y. Wang, G.S. Wang, P.G. Yin, Excellent electromagnetic wave absorbing properties of two-dimensional carbon-based nanocomposite supported by transition metal carbides Fe_3_C. Carbon **162**, 438–444 (2020). https://doi.org/10.1016/j.carbon.2020.02.031

S4 F. Pan, Z. Liu, B. Deng, Y. Dong, X. Zhu et al., Magnetic Fe_3_S_4_ LTMCs micro-flowers @ wax gourd aerogel-derived carbon hybrids as efficient and sustainable electromagnetic absorber. Carbon **179**, 554–565 (2021). https://doi.org/10.1016/j.carbon.2021.04.053

S5 J.B. Cheng, H.B. Zhao, A.N. Zhang, Y.Q. Wang, Y.Z. Wang et al., Porous carbon/Fe composites from waste fabric for high-efficiency electromagnetic wave absorption. J. Mater. Sci. Technol. **126**, 266–274 (2022). https://doi.org/10.1016/j.jmst.2022.02.051

S6 W. Huang, Q. Qiu, X. Yang, S. Zuo, J. Bai, Ultrahigh density of atomic CoFe-electron synergy in noncontinuous carbon matrix for highly efficient magnetic wave adsorption. Nano-Micro Lett. **14**, 96 (2022). https://doi.org/10.1007/s40820-022-00830-8

S7 Z. Yang, T. Wang, J. Wang, Z. Luo, Q. Zhang et al., Heterogeneous N-doped carbon composite NiSe_2_–FeSe double-shell hollow nanorods for tunable and high-efficient microwave attenuation. Carbon **201**, 491–503 (2023). https://doi.org/10.1016/j.carbon.2022.09.023

S8 J. Su, Q. Ma, L. Que, H. Jiang, X. Xu et al., Multi-componential metal intercalated graphite hybrids synthesized by co-intercalation polymerization towards high-efficient microwave absorptions. Nano Res. **16**, 6369–6379 (2023). https://doi.org/10.1007/s12274-023-5483-7
